# Essential Oils and Plant-Derived Volatile Compounds in the Treatment of Oral Candidiasis: A Systematic Review of Randomized Controlled Trials in Denture Stomatitis

**DOI:** 10.3390/jcm15218617

**Published:** 2026-11-05

**Authors:** Barbara Lipka, Małgorzata Kępa, Maciej Łopaciński, Aleksandra Warakomska, Maciej Dobrzyński, Marek Ziętek, Dariusz Skaba, Rafał Wiench

**Affiliations:** 1 Department of Periodontal Diseases and Oral Mucosa Diseases, Faculty of Medical Sciences in Zabrze, Medical University of Silesia in Katowice, 40-055 Katowice, Poland; mlopacinski@sum.edu.pl (M.Ł.); awarakomska@sum.edu.pl (A.W.); dskaba@sum.edu.pl (D.S.); rwiench@sum.edu.pl (R.W.); 2 Department of Microbiology, Faculty of Pharmaceutical Sciences in Sosnowiec, Medical University of Silesia, 41-200 Sosnowiec, Poland; mkepa@sum.edu.pl; 3 Department of Pediatric Dentistry and Preclinical Dentistry, Wrocław Medical University, Krakowska 26, 50-425 Wrocław, Poland; maciej.dobrzynski@umw.edu.pl; 4 Department of Experimental Dentistry, Wrocław Medical University, Krakowska 26, 50-425 Wrocław, Poland; marek.zietek@umw.edu.pl

**Keywords:** essential oils, oral candidiasis, denture stomatitis, *Candida albicans*, phytotherapy, plant-derived volatile compounds, antifungal therapy, antifungal resistance, randomized controlled trials

## Abstract

**Background:** Denture stomatitis, the most common form of oral candidiasis among denture wearers, remains highly prevalent, and antifungal resistance is an emerging concern. Essential oils and plant-derived volatile compounds show broad-spectrum antifungal activity in vitro, generating interest as complementary therapies. This review evaluated the clinical efficacy, mycological efficacy, and safety of these compounds versus conventional antifungals or placebo, based solely on randomized controlled trials (RCTs). **Methods:** Following PRISMA 2020 guidelines and PROSPERO registration (CRD420261473618), PubMed, Embase, Scopus, and Cochrane CENTRAL were searched on 20 July 2026 for English-language RCTs published up to 30 June 2026 and evaluating topical essential oils or volatile compounds against a conventional antifungal or placebo comparator in oral candidiasis. Two reviewers independently conducted screening, extraction and RoB 2 risk-of-bias assessment. Due to heterogeneity, findings were synthesized narratively. **Results:** Eight RCTs (347 participants), all in denture stomatitis patients, met inclusion criteria, evaluating oils from *Zataria multiflora*, *Cinnamomum zeylanicum*, *Melaleuca alternifolia*, *Pelargonium graveolens*, *Satureja hortensis*, *Artemisia sieberi*, and clove/cinnamon extracts. In active-comparator trials, no statistically significant differences in clinical outcomes were detected, but these trials were small and underpowered and none defined a non-inferiority margin; mycological efficacy varied, sometimes favoring nystatin or miconazole. Both placebo-controlled trials favored essential oil gels for clinical and mycological recovery. Adverse effects were inconsistently and mostly non-systematically reported; one trial reported vertigo, nausea and vomiting in the essential-oil group. Overall risk of bias was low in three trials and raised some concerns in five for both outcomes. **Conclusions:** The evidence for essential oils in denture stomatitis is preliminary and insufficient to support replacing conventional antifungal therapy; the findings cannot be extrapolated to other forms of oral candidiasis. Safety data are insufficient to draw firm conclusions. Standardized formulations and larger, well-designed RCTs with longer follow-up and safety assessment are needed.

## 1. Introduction

Oral candidiasis is among the most common fungal infections in humans, and denture-associated stomatitis (chronic erythematous or atrophic candidiasis) is generally regarded as its most frequent clinical form among removable-denture wearers [1]. Reported prevalence estimates for denture stomatitis range from approximately 20% to 67% among denture wearers [2,3]. The condition typically presents as erythema and edema of the mucosa in direct contact with the fitting surface of the denture, graded clinically according to the Newton classification, and may be accompanied by burning or discomfort [4].

The etiology of denture stomatitis is multifactorial, but the central driver is the formation of a *Candida*-rich microbial biofilm on the intaglio surface of the prosthesis and the underlying mucosa, promoted by poor denture and oral hygiene, continuous or nocturnal denture wear, ill-fitting appliances, xerostomia, and the porosity of denture base material [5]. *Candida albicans* remains the predominant species, although non-*albicans* species are increasingly implicated [6]. The persistence of these biofilms on denture surfaces sustains fungal colonization and contributes to disease recurrence after treatment, so that effective management requires both mucosal antifungal therapy and denture disinfection alongside hygiene measures [7].

Conventional management relies on topical or systemic antifungal agents, principally the polyene nystatin and the azoles miconazole, clotrimazole, and fluconazole. However, antifungal resistance is an increasing concern for these drugs, although its relevance varies by clinical setting. Resistance in *Candida* species arises through multiple mechanisms, including efflux-pump overexpression, mutation or overexpression of the ergosterol-biosynthesis target enzyme (ERG11/lanosterol 14-α-demethylase), biofilm-mediated drug tolerance, and adaptive stress responses [8,9]. This is compounded by an epidemiological shift toward intrinsically less susceptible non-*albicans* species and the emergence of multidrug-resistant organisms such as *Candida auris* [6,10]. Most of these resistance data concern systemic azoles, particularly fluconazole, and non-albicans species, largely in invasive infection or in immunocompromised hosts. Denture stomatitis, in contrast, is predominantly treated with topical nystatin or miconazole, and the clinical relevance of resistance in this setting is less well established; the main therapeutic problem is recurrence after treatment from denture-associated biofilm. Resistance concerns, together with this recurrence, have nevertheless intensified interest in complementary and alternative antimicrobial strategies for denture stomatitis.

Essential oils are complex mixtures of volatile secondary metabolites, composed mainly of terpenes, terpenoids, and, in some oils, phenylpropanoids, typically containing 20–60 constituents at widely differing concentrations [11]. Their composition may vary according to plant species, chemotype, plant part, extraction method, and storage conditions; essential oils should therefore not be regarded as chemically uniform interventions [12,13]. This chemical diversity underlies a broad spectrum of biological activity, including antibacterial, antifungal, antiviral, anti-inflammatory, and antioxidant effects [14]. Supercritical CO_2_ extracts are distinguished from essential oils because they may contain both volatile and non-volatile constituents. The extracts evaluated in this review were included because of their volatile constituents.

The antimicrobial and antifungal activity of essential oils and their isolated constituents is attributed to multiple, simultaneous cellular targets. Owing to their lipophilicity, terpene and phenolic components partition into and disrupt the microbial cell membrane, increasing permeability and causing extravasation of ions and cytoplasmic contents, leading to loss of membrane integrity and cell lysis [14,15]. Against fungi, essential oils additionally inhibit ergosterol biosynthesis, interfere with cell-wall components such as β-glucans and chitin, disrupt ion homeostasis, and induce oxidative stress [16]. Because these effects engage several targets at once, resistance is thought to develop more slowly than to single-target conventional antifungals, and several constituents show synergy with existing agents [16]. Phenol- and terpenoid-rich oils, including those from *Cinnamomum*, *Thymus*, *Origanum*, and *Syzygium*, and constituents such as thymol, carvacrol, eugenol, and cinnamaldehyde, are among the most active against *Candida* spp. [17].

Essential oils have been investigated in a range of medical settings, including dermatological conditions, topical fungal infections, acne, and the decolonization of methicillin-resistant *Staphylococcus aureus* (MRSA), although the quality and certainty of the available clinical evidence remain variable [18]. Their antimicrobial potential has also stimulated interest in oral health applications. In the context of oral candidiasis and denture stomatitis, essential oils may offer a complementary approach because of their reported activity against *Candida* spp. and related biofilms; however, most supporting data derive from in vitro work, and their clinical effectiveness and safety remain insufficiently established [19].

Given the prevalence of denture stomatitis, the emerging concern of antifungal resistance, the recurrent nature of denture stomatitis, and the growing but heterogeneous literature on plant-derived volatile compounds, a rigorous appraisal of the clinical evidence is warranted. Therefore, this systematic review aimed to evaluate the clinical and mycological outcomes, safety, and tolerability of essential oils and other plant-derived volatile compounds, compared with conventional antifungal therapy or placebo, in patients with oral candidiasis, restricting the analysis to randomized controlled trials.

## 2. Materials and Methods

### 2.1. PICO Framework

Based on the PICO framework [20], the focused question of the present systematic review was: in patients with oral candidiasis (Population), do essential oils or plant-derived volatile compounds (Intervention), compared with conventional antifungal therapies or placebo (Comparison), improve clinical outcomes such as clinical cure, mycological outcomes such as reduction or eradication of *Candida* spp., symptom relief, and recurrence rates (Outcome)?

### 2.2. Search Strategy

**Figure 1:**
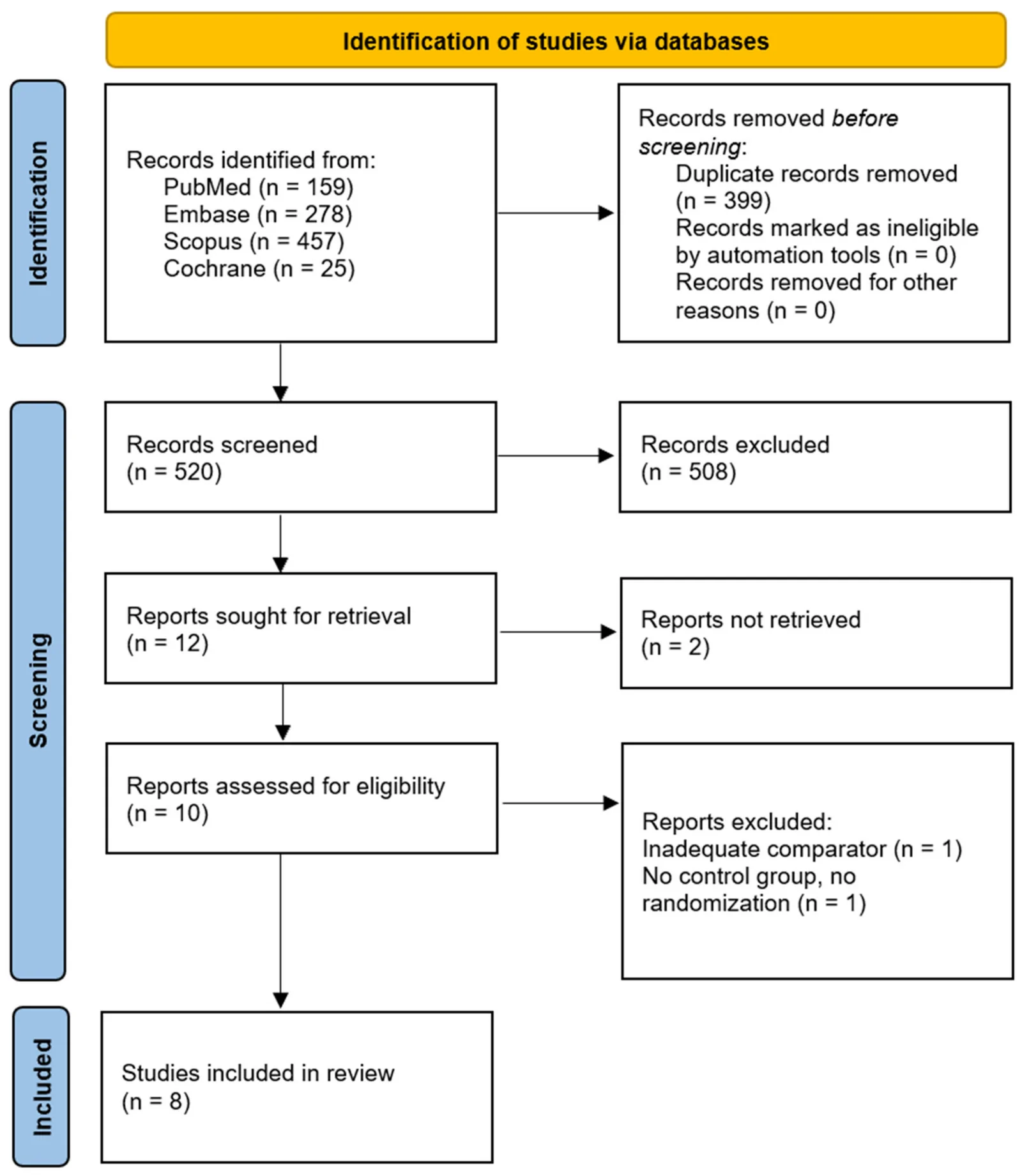
PRISMA 2020 flow diagram.

This systematic review was registered in PROSPERO (CRD420261473618) on 8 August 2026 and conducted in accordance with the PRISMA 2020 statement [21]. The completed PRISMA 2020 checklist is provided in the Supplementary Materials. PubMed, Embase, Scopus, and Cochrane CENTRAL were independently searched on the single date of 20 July 2026 by B.L. and M.K., using database-specific combinations of controlled vocabulary terms (MeSH/Emtree, where applicable) and keywords related to oral candidiasis and essential oils (Table S1). The detailed, line-by-line search strategy for Cochrane CENTRAL is provided in Table S2. Eligibility was restricted to English-language studies published up to 30 June 2026. This cutoff refers to publication date, not database entry date, and was chosen to include studies published through the last complete calendar month preceding the search. “Snowballing” was performed; however no additional publications were identified. After duplicate removal, titles and abstracts were independently screened by B.L. and M.K. against the predefined eligibility criteria. Full texts of potentially eligible records were subsequently independently assessed by the same reviewers, with reasons for exclusion recorded (Table 1). For trial registrations identified through Cochrane CENTRAL, bibliographic databases were searched for corresponding full-text publications reporting completed trial results. Registrations without a retrievable publication were excluded from the synthesis. Disagreements at either stage were resolved through discussion or, when necessary, by arbitration of a third reviewer (R.W.). Inter-reviewer agreement during title and abstract screening was assessed using Cohen’s kappa (κ = 0.82). Studies meeting the eligibility criteria by consensus proceeded to data extraction (Figure 1, PRISMA 2020 flow diagram). There were no deviations from the registered protocol.

### 2.3. Selection of the Studies

Specific eligibility criteria were established collectively by all reviewers (B.L., M.K., and R.W.) and are presented in Table 1. Eligible studies were randomized controlled trials evaluating essential oils or other plant-derived volatile compounds for the treatment of oral candidiasis in comparison with a conventional antifungal agent or placebo.

Borderline eligibility decisions are reported here for transparency.

(1) Iyer et al. evaluated single emulgel containing clove and cinnamon supercritical CO_2_ extracts with identified volatile constituents, including eugenol and cinnamaldehyde. The formulation was retained because both extracts fell within the review’s scope, whereas the combination-product exclusion criterion concerned mixtures with other active herbal preparations outside this scope. Findings were attributed to the combined formulation, as the contribution of each extract could not be isolated.

(2) The 1% *Artemisia sieberi* mouthwash in Sefidgar et al. was suggested in Section 4 as an essential oil, but its extraction method and composition were not reported. (3) The commercial product used by Gonoudi et al. Gastroli® (Bridge Essence, Kashan, Iran) was treated as a *Zataria multiflora* essential-oil product. (4) Catalán et al. incorporated the essential oil into a tissue conditioner, so the intervention also has the character of a material (device) intervention; the trial was retained because the essential oil was the experimental variable compared with a nystatin-loaded and an unloaded conditioner, but it is interpreted separately from the mucosal formulations and with caution.

### 2.4. Risk of Bias Assessment

Risk of bias was assessed using version 2 of the Cochrane Risk of Bias tool (RoB 2) for the primary clinical outcome and the secondary mycological outcome at the end of treatment [22]. The clinical outcome was defined as resolution or improvement of denture stomatitis, whereas the mycological outcome was defined as reduction or eradication of *Candida* spp. Two reviewers (B.L. and M.K.) independently assessed each study across five domains: bias arising from the randomization process, bias due to deviations from intended interventions, bias due to missing outcome data, bias in measurement of the outcome, and bias in selection of the reported result. Discrepancies were resolved through discussion, with a supervisor (R.W.) consulted when necessary to reach a final judgement. Responses to the signalling questions and the supporting information for every domain-level judgement are provided in Table S3. Overall judgements followed the RoB 2 algorithm, under which a high-risk judgement in any domain results in high risk of bias overall.

### 2.5. Data Extraction

Data were independently extracted by two reviewers (B.L. and M.K.) using a predefined data extraction form. Discrepancies were resolved through discussion, with a supervisor (R.W.) consulted when necessary. Extracted data included author and year of publication, country, study design and blinding, participant characteristics, sample size calculation and number of participants, clinical condition, characteristics of the plant-derived volatile substance and formulation, intervention and comparator details, treatment regimen and duration, denture management during treatment, clinical and mycological outcome measures, assessment time points and follow-up duration, adverse events, tolerability and patient reported outcomes, and key findings. Where data were unclear or missing, this was recorded and reported narratively.

### 2.6. Data Synthesis

Owing to substantial heterogeneity in interventions, comparators, treatment protocols, outcome measures, and assessment time points, meta-analysis was not performed. Findings were synthesised narratively and summarised according to study characteristics, intervention and comparator, clinical and mycological outcomes, follow-up, and safety and tolerability outcomes.

## 3. Results

### 3.1. Study Selection

The initial search across four databases identified 919 records. After removal of 399 duplicate records, 520 records remained for title and abstract screening. Of these, 508 records were excluded. Full texts of 12 reports were sought for retrieval; however, two reports could not be retrieved. The two records for which corresponding full-text publications could not be retrieved were trial registrations identified through Cochrane CENTRAL: CTRI/2024/02/062591 (CN-02682364) and IRCT201507259014N72 (CN-01805903). Searches of bibliographic databases did not identify corresponding full-text publications reporting trial results. It also remained unclear whether these trials had been completed at the time of the search. The investigators were not contacted.

Ten full-text reports were assessed for eligibility. Two reports were excluded: Vazquez et al. because the comparator was inadequate, as both groups received oil-based mixtures and only the vehicle differed; and Jandourek et al. because the study had no control group and did not use randomization [23,24].

In total, eight randomized controlled trials (RCTs) meeting the predefined eligibility criteria and published up to 30 June 2026 were included in this systematic review. The PRISMA flow diagram is presented in Figure 1 [21]. The main characteristics of the included studies are presented in Table 2.

### 3.2. Risk of Bias Assessment

**Figure 2:**
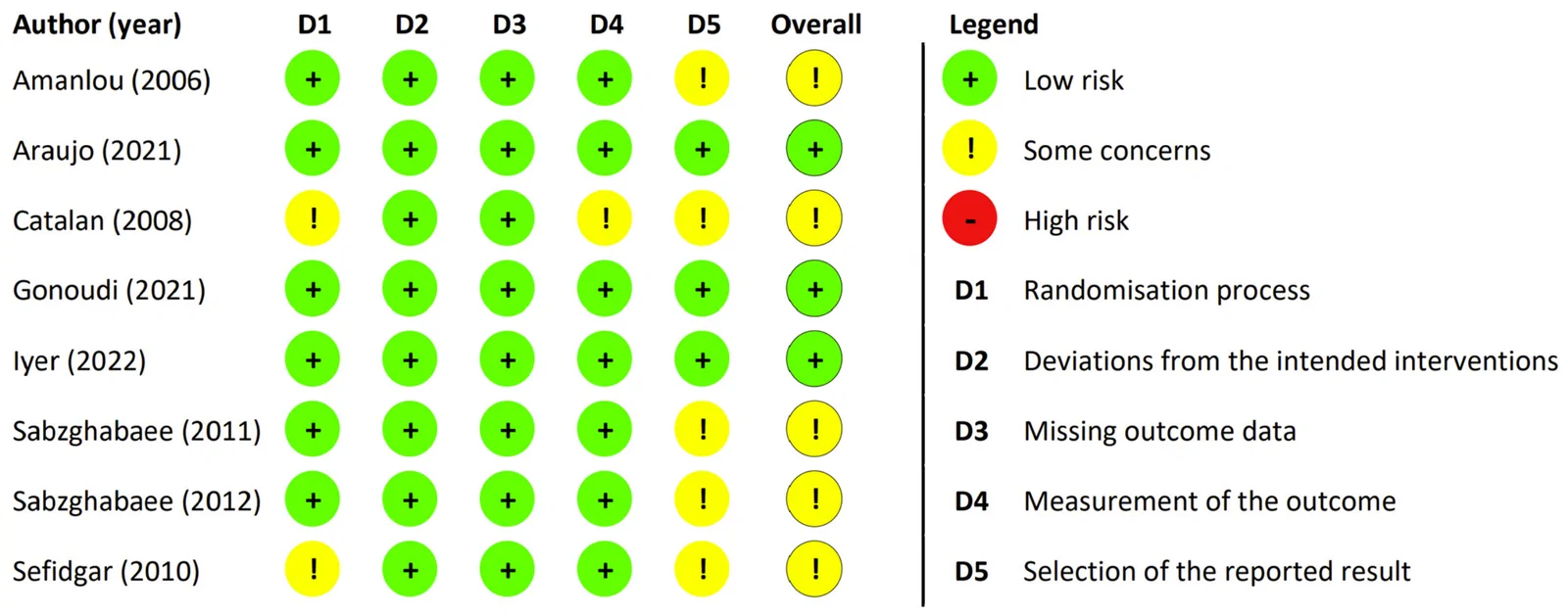
Risk-of-bias assessment for the primary clinical outcome at the end of treatment. D—domain; D1, randomisation process; D2, deviations from the intended interventions; D3, missing outcome data; D4, measurement of the outcome; D5, selection of the reported result (the risk that the reported result was chosen from several possible measurements or analyses). Studies evaluated [25,26,26,27,28,29,30,31,32].

Risk of bias was assessed separately for the primary clinical outcome and the secondary mycological outcome at the end of treatment using the Cochrane Risk of Bias 2 (RoB 2) tool [22]. For both outcomes, three studies were judged to be at low overall risk of bias (Araújo, Gonoudi, and Iyer), while five raised some concerns (Amanlou, Catalán, both Sabzghabaee trials, and Sefidgar). No study was judged to be at high overall risk of bias (Figure 2 and Figure S1).

Concerns arose mainly from selection of the reported result (D5), with additional concerns regarding the randomisation process (D1) in Catalán (2008) and Sefidgar (2010). Overall judgements were identical across outcomes [27,32]. Domain-level judgements differed only for Catalán (2008), for which measurement of the outcome (D4) raised some concerns for the clinical outcome but was judged to be at low risk for the mycological outcome [27]. All remaining studies were judged to be at low risk in D4 for both outcomes. Responses to the signalling questions and the supporting information for every domain-level judgement are provided in Table S3.

### 3.3. General Characteristics of the Included Studies

The general characteristics of the eight included studies, including publication year, country, study design, and blinding, are presented in Table 2.

**Table 2:** General characteristics of included studies.

First Author(s) and Reference	Year	Country	Study Design	Blinding
Amanlou et al. [25]	2006	Iran	RCT	Double-blind
Araújo et al. [26]	2021	Brazil	RCT	Double-blind
Catalán et al. [27]	2008	Chile	RCT	Not reported
Gonoudi et al. [28]	2021	Iran	RCT	Single-blind
Iyer et al. [29]	2022	India	RCT	Double-blind
Sabzghabaee et al. (*P. graveolens*) [30]	2011	Iran	RCT	Double-blind
Sabzghabaee et al. (*S. hortensis*) [31]	2012	Iran	RCT	Double-blind
Sefidgar et al. [32]	2010	Iran	RCT	Double-blind

RCT—randomized controlled trial.

### 3.4. Characteristics of Participants in the Included Studies

Participant characteristics are presented in Table 3. Although the review did not restrict inclusion to denture-related oral candidiasis, all included studies evaluated patients with denture stomatitis. Most studies included users of complete, predominantly maxillary dentures; however, denture type was not consistently reported across trials.

### 3.5. Characteristics of Plant-Derived Volatile Substances and Preparations Used in the Included Studies

Table 4 presents the characteristics of plant-derived volatile preparations used in the included studies, including plant source, plant part, extraction method or source, reported chemical composition, clinical concentration, and pharmaceutical formulation.

### 3.6. Interventions and Protocols in Included RCTs

The interventions and treatment protocols used in the included studies are summarised in Table 5, and show substantial heterogeneity. With the exception of Catalán et al. [27], all studies used a conventional two-arm, parallel-group RCT design [25,26,28,29,30,31,32]. Catalán et al. used a three-arm design comparing two active substances incorporated into the same tissue conditioner with the tissue conditioner alone as the control.

In the experimental groups, essential oils were used in different formulations, including gels, mouthwashes, solutions, sprays, and an emulgel [25,26,27,28,29,30,31,32]. In most studies, the EO-containing product was administered directly to the mucosa [25,26,28,29,30,31,32]. Catalán et al. was the only study in which the EO was incorporated into a Coe-Comfort tissue conditioner, which was applied to the intaglio surface of the maxillary denture as a liner and worn continuously [27].

Regarding comparators, six of the eight included studies used conventional antifungal therapy, including nystatin, miconazole, or clotrimazole [25,26,27,28,29,32]. In contrast, the two studies by Sabzghabaee et al. compared EO-containing gels with a placebo gel [30,31]. Treatment duration was predefined in most studies and ranged from 12 to 21 days; however, in Sefidgar et al., treatment was continued until clinical resolution rather than for a fixed duration [32].

Denture management during treatment was also heterogeneous. In addition to standard denture hygiene instructions, active antifungal treatment was applied directly to the denture surface in the studies by Amanlou et al., Araújo et al., Catalán et al., and both studies by Sabzghabaee et al. [25,26,27,30,31]. In Sefidgar et al., denture use was discontinued during treatment [32].

### 3.7. Main Study Outcomes

Clinical and mycological outcome measures, assessment time points, and the main clinical and mycological findings are presented in Table 6. Safety, tolerability, and patient-reported outcomes were reported inconsistently, assessed using heterogeneous methods, and evaluated in only a few studies; therefore, they were synthesized narratively rather than tabulated.

#### 3.7.1. Clinical and Mycological Outcome Measures and Assessment Time Points

Clinical outcome assessment varied across the included trials, and is presented in Table 6. Quantitative measurement of the palatal erythema surface area was used by Amanlou et al. and Gonoudi et al.; where the affected area was outlined and measured using a 5 mm squared transparent grid [25,28]. Araújo et al. and Iyer et al. assessed clinical changes using the Newton classification [26,29]. Catalán et al. applied a four-point, author-defined scale of palatal inflammation [27]. In both studies by Sabzghabaee et al., treatment response was classified as complete, partial, or no clinical recovery, based on changes in palatal erythema and fungal colony counts [30,31]. Sefidgar et al. used a distinct approach, recording complete clinical resolution of denture stomatitis rather than baseline severity or changes in disease intensity [32].

Mycological assessment was also heterogeneous (Table 6). Amanlou et al. and Iyer et al. reported overall *Candida* colony counts or CFU without species reporting [25,29]. Araújo et al. quantified *Candida* spp. CFU and performed species identification, reporting *C. albicans*, *C. tropicalis*, *C. krusei*, and *C. glabrata* [26]. Catalán et al. and Gonoudi et al. specifically evaluated *C. albicans*; in the latter study, species identification was confirmed using a germ-tube test [27,28]. Both studies by Sabzghabaee et al. assessed fungal colony counts, with *C. albicans* confirmed in the study populations [30,31]. Sefidgar et al. used a semiquantitative *Candida* colony-growth score and identified *C. albicans*, *C. tropicalis*, and *C. krusei* [32].

Assessment schedules also differed between trials (Table 6). Outcomes were measured at baseline and at one or more time points during treatment. Only Amanlou et al., Sabzghabaee et al. (*S. hortensis*), and Sefidgar et al. included post-treatment follow-up, which extended to 14 days after treatment in Amanlou et al., 7 days in the study by Sabzghabaee et al., and approximately 1 week after clinical resolution in Sefidgar et al. [25,31,32]. No included study reported long-term follow-up for clinical recurrence. Symptom relief and recurrence, although specified in the PICO framework, were not reported as defined outcomes in any included trial; the only post-treatment follow-up was short (up to 14 days) and was not designed to assess recurrence.

#### 3.7.2. Main Clinical Outcomes

The clinical outcomes are presented in Table 6. Across the included trials, essential-oil-based interventions were generally associated with clinical improvement, including reductions in palatal erythema or inflammation, improvement according to the Newton classification, and clinical resolution of denture stomatitis [25,26,27,28,29,30,31,32]. In trials comparing EO formulations with active antifungal treatments, no statistically significant differences in clinical outcomes between groups were generally detected; however, these trials were small (9–21 participants per arm) and none defined a non-inferiority margin, so the absence of a significant difference does not demonstrate equivalence [25,26,27,28,29,32]. No statistically significant between-group differences were reported by Amanlou et al., Araújo et al., Gonoudi et al., Catalán et al., or Sefidgar et al. [25,26,27,28,32]. In both placebo-controlled trials by Sabzghabaee et al., *Pelargonium graveolens* and *Satureja hortensis* gels achieved greater clinical recovery than placebo [30,31]. The clove/cinnamon emulgel evaluated by Iyer et al. was associated with a numerically higher cure rate than clotrimazole gel at day 21 (47.6% vs. 42.9%; 10/21 vs. 9/21) [29].

#### 3.7.3. Main Mycological Outcomes

The mycological outcomes are presented in Table 6. Across the included trials, EO-based interventions were generally associated with reductions in fungal burden. However, mycological outcomes were assessed using heterogeneous methods, including colony counts, CFU, and semiquantitative colony-growth scores [25,26,27,28,29,30,31,32]. In active-comparator trials, both intervention and comparator groups generally showed mycological improvement [25,26,27,28,29,32]. Thus, findings varied according to the comparator and sampling site. In Araújo et al., nystatin showed greater reductions in *Candida* counts than *C. zeylanicum* on both oral-mucosa (89% vs. 61%) and denture surfaces (83% vs. 33%) [26]. Similarly, Amanlou et al. found miconazole to be more effective than *Zataria multiflora* gel in reducing denture-surface colony counts [25]. Iyer et al. reported a greater reduction in denture surface *Candida* CFU with the clove/cinnamon emulgel than with clotrimazole gel [29]. In contrast, Gonoudi et al. and Sefidgar et al. found no significant between-group differences in mycological outcomes [28,32]. Catalán et al. likewise reported no difference between *M. alternifolia* and nystatin at the final assessment, while both active treatments were superior to the tissue-conditioner-only control [27]. In placebo-controlled trials by Sabzghabaee et al., *P. graveolens* and *S. hortensis* gels produced significantly greater reductions in fungal colony counts than placebo [30,31].

#### 3.7.4. Summary of Findings by Outcome Type

Clinical outcome: in the two placebo-controlled trials (n = 160), EO gels achieved greater clinical recovery than placebo; in the six active-comparator trials (n = 187), no statistically significant differences in clinical outcomes were detected in small, underpowered trials. Mycological outcome: both placebo-controlled trials showed greater reductions in fungal counts with EO gel; among active-comparator trials, nystatin or miconazole achieved greater reductions in two (Araújo, Amanlou, mainly on denture surfaces), no between-group difference was found in three (Gonoudi, Sefidgar, Catalán), and a greater denture-surface reduction with the emulgel than with clotrimazole was reported in one (Iyer). Safety: data were sparse and non-systematic (Section 3.7.5). Main dimensions of heterogeneity (Table 4, Table 5 and Table 6): seven plant sources; concentrations from 0.05% to 1% (or equivalent); gel, mouthwash, solution, emulgel and tissue-conditioner formulations; nystatin, miconazole, clotrimazole or placebo comparators; treatment durations of 12–21 days or until resolution; and clinical outcomes defined by area of erythema, ordinal scores, composite recovery or resolution.

#### 3.7.5. Safety, Tolerability, and Patient-Reported Outcomes

Safety, tolerability, and patient-reported outcomes were inconsistently reported across the included trials and were therefore not included in Table 6. The available safety data are summarised study by study in Table S4. Amanlou et al., Araújo et al., and Sabzghabaee et al. (*S. hortensis*) described adverse-effect assessment in their Methods sections, although the extent of assessment and reporting varied [25,26,31]. In the remaining studies, tolerability, taste, or adverse effects were reported inconsistently, often only in the Results or Discussion sections; several studies did not report these [27,28,29,30,32]. Araújo et al. provided the most detailed prospective assessment of patient-reported outcomes [26]. Undesirable effects were reported by 16.7% of participants in the *Cinnamomum zeylanicum* EO group: slight numbness in two participants and slight burning in one participant. In the nystatin group, tongue sensitivity was reported by 5.6% of participants. Taste was rated as pleasant by 83.3% of participants in the EO group and by 61.1% of participants in the nystatin group. Amanlou et al. assessed local adverse reactions and questioned participants about adverse effects at each visit. [25]. In the *Zataria multiflora* group, itching, burning, vertigo, nausea, vomiting, and bad taste were reported. The original publication states a frequency of 59.3%, but provides no numerator or explanation of the denominator; this percentage cannot be reconciled with the reported group size of 12 participants. In the miconazole group, 50% of participants reported burning, vomiting, or bad taste. Iyer et al. assessed taste acceptability using a hedonic scale and reported greater acceptability of the clove/cinnamon emulgel than the commercial clotrimazole gel [29]. The authors also stated that no side effects were observed; however, the method used to assess safety or adverse effects was not reported. In the Satureja hortensis trial, patients were assessed for local adverse reactions and questioned about adverse effects on days 14 and 21; Sabzghabaee et al. reported that the spicy odor of the *Satureja hortensis* EO gel was the only minor complaint, with no other reportable side effects [31]. In the study of *Pelargonium graveolens* gel by Sabzghabaee et al. [30], no reportable side effects were reported. Sefidgar et al. reported no complaints in the *Artemisia sieberi* mouthwash group; the taste was described as desirable [32]. One participant in the nystatin group reported an unpleasant taste. Safety, tolerability, and patient-reported outcomes were not reported by Catalán et al. or Gonoudi et al. [27,28].

## 4. Discussion

### 4.1. Results in the Context of Other Evidence

The present review included eight RCTs evaluating essential-oil-based interventions for oral candidiasis [25,26,27,28,29,30,31,32]. Despite substantial heterogeneity in botanical source, formulation, concentration, administration regimen, and comparator, the included studies generally reported favourable clinical and/or mycological outcomes with EO-based interventions. These findings provide preliminary clinical support for the anticandidal potential of plant-derived volatile compounds and warrant interpretation alongside the broader preclinical and limited clinical evidence.

A substantial body of research has investigated the anticandidal activity of essential oils and their isolated constituents against *Candida* spp.; however, most evidence is derived from in vitro studies, with limited data from animal models and human trials. Ferreira et al. and da Silva et al. systematically reviewed 26 and 38 studies, respectively, evaluating the in vitro anti-*Candida* activity of various essential oils [33,34]. Both reviews highlighted substantial heterogeneity, precluding firm conclusions. Nevertheless, the available evidence supports the in vitro anti-*Candida* potential of *Cinnamomum zeylanicum* and *Melaleuca alternifolia*, both of which were evaluated in the present review. Evidence from animal models is limited, with two mouse studies of *M. alternifolia* reporting reduced oral *C. albicans* burden, including with an azole-resistant strain [35,36]. Clinical evidence is even more limited. Two studies excluded during full-text eligibility assessment evaluated *M. alternifolia* oral solution in patients with AIDS and fluconazole-refractory oral or oropharyngeal candidiasis [23,24]. After four weeks, clinical responses were reported in 8 of 12 evaluable patients in the study by Jandourek et al. and in 60% of patients in the study by Vazquez et al. [23,24].

Although this review aimed to evaluate essential-oil-based interventions for oral candidiasis regardless of clinical subtype, all eligible studies investigated denture stomatitis. This reflects the clinical relevance of the condition, which affects approximately 20–67% of complete and partial acrylic denture wearers worldwide [2,3]. The persistence of *Candida* biofilms on denture surfaces may contribute to disease recurrence by sustaining fungal colonisation after treatment [37,38]. Several included RCTs incorporated denture disinfection into the treatment protocol by applying the active formulation to both the oral mucosa and denture surface [25,26,30,31]. Methodologically, these cointerventions may make it difficult to isolate the effect of the EO formulation on oral candidiasis. Clinically, however, they are justified, as denture disinfection alongside mucosal treatment is recommended in the management of denture stomatitis [39,40]. The challenge of eliminating *Candida* from denture surfaces has also been explored in vitro using essential-oil-based solutions on denture base materials, particularly polymethyl methacrylate (PMMA). In a review of 12 essential oils, Carvalho-Silva et al. found that several oils, including *C. zeylanicum* and *M. alternifolia*, reduced *Candida* biofilm on dental acrylic resin [19]. *Zataria multiflora* essence showed in vitro efficacy comparable to nystatin on contaminated acrylic-resin plates [41]. *Artemisia sieberi* extract has also been examined on digitally fabricated denture-base acrylic [42,43].

Safety and tolerability are important considerations for clinical use. Among the included RCTs, Amanlou et al., Araújo et al., and Sabzghabaee et al. (*S. hortensis*) described adverse-effect assessment, whereas Iyer et al. assessed taste acceptability without describing a clinical adverse-event assessment method. [25,26,29,31]. Evidence beyond these trials is limited. Notably, Amanlou et al. reported vertigo, nausea and vomiting in the *Zataria multiflora* group, and most other trials did not assess adverse effects prospectively; the safety of these preparations therefore cannot be considered established. Oliveira et al. reported no adverse clinical signs following use of a *Cinnamomum zeylanicum* essential-oil mouthwash in 15 healthy denture wearers, apart from mild, transient burning in one participant [44]. An Orabase formulation by Alves et al. containing cinnamaldehyde, a major constituent of *C. zeylanicum* oil, was likewise generally well tolerated, with only mild, transient burning [45]. A systematic review by Kairey et al. found that low-concentration oral *Melaleuca alternifolia* formulations were generally associated with mild, transient effects, including burning, unpleasant taste, and occasional nausea [46]. Nevertheless, safety data on essential oils for denture stomatitis and oral candidiasis remain limited because of study heterogeneity, small sample sizes, and inconsistent adverse-event reporting. Without systematic safety assessment, it is difficult to recommend essential oils even as adjunctive therapy, particularly for mucosal application, where irritation, sensitisation or systemic absorption may occur. No usable data on symptom relief or recurrence rates were available from the included trials; therefore, these outcomes could not be synthesised.

Overall, evidence from in vitro studies, animal models, and additional human studies is consistent with the findings of the included RCTs and supports the potential of EO-based interventions for oral candidiasis, particularly denture-related candidiasis. Nevertheless, substantial heterogeneity in the essential oils and formulations evaluated, as well as in study protocols, was evident across both the included RCTs and the studies discussed above. This heterogeneity hindered firm conclusions regarding efficacy and may also have contributed to the limited number of available RCTs. Together, these findings highlight the need for further standardised in vitro studies and well-designed clinical trials, with particular emphasis on the assessment of safety and tolerability.

### 4.2. Limitations of the Evidence

The findings of this review should be interpreted in light of important limitations of the included evidence. Only eight RCTs with relatively small sample sizes were identified. The interventions were heterogeneous with respect to the essential oils or plant-derived volatile extracts evaluated, concentrations, formulations, treatment regimens, and durations. In several trials, incomplete reporting of chemical composition or formulation base precluded assessment of pharmacological equivalence. Comparators and cointerventions also varied. Catalán et al. incorporated the essential oil into a tissue conditioner, an intervention that also has the character of a material (device) intervention, which limits comparability with the mucosal formulations. Although no trial was judged to be at high overall risk of bias for either assessed outcome, most trials raised some concerns. Most studies used conventional antifungal agents, whereas two used placebo controls. Denture management was inconsistent: some trials applied the intervention to both the oral mucosa and denture surface, while others provided hygiene instructions, modified denture use, or did not report denture management procedures. These differences may have affected clinical and mycological outcomes and limited isolation of the effect attributable to the EO formulation. Clinical assessment varied substantially in precision, ranging from quantitative measurement of palatal erythema with a transparent grid to ordinal classifications, author-defined inflammation scores, or overall clinical resolution. Mycological assessment was relatively more consistent, as most studies quantified fungal burden; however, methods included colony counts, CFU measurements, and semiquantitative growth scores. This heterogeneity precluded meta-analysis and limits direct comparisons between interventions. Follow-up, where reported, was short, and no study assessed long-term recurrence. Safety, tolerability, and patient-reported outcomes were largely absent from the included trials, with only a small number of studies assessing them prospectively. Therefore, despite generally favourable findings, the available evidence does not support firm conclusions regarding comparative efficacy, optimal formulation or regimen, long-term effectiveness, or safety of EO-based interventions for denture stomatitis.

### 4.3. Limitations of the Review Process

A meta-analysis was not feasible because of substantial heterogeneity in the interventions, comparators, co-interventions, outcome definitions, measurement methods, and assessment time points. Therefore, the findings were synthesised narratively, which does not provide a pooled estimate of treatment effect and limits the precision of conclusions. In addition, the review was restricted to English-language publications and to four bibliographic databases. The language restriction may have excluded relevant studies published in other languages and introduced language bias. Regional databases (e.g., SID and Magiran), trial registries (ClinicalTrials.gov, ICTRP, and IRCT), and grey literature were not searched, which is an important limitation because five of the eight included trials were conducted in Iran; relevant completed or ongoing trials and unpublished results may therefore have been missed. Full-text publications corresponding to two trial registrations identified through Cochrane CENTRAL could not be retrieved, and their completion status at the time of the search could not be established. The investigators were not contacted, leaving uncertainty about the availability of additional relevant evidence.

The certainty of the evidence was not formally assessed using GRADE, which limits the transparency of judgements about the strength of the evidence. Publication bias and small-study effects were not assessed using funnel plots or statistical tests because few studies were available within each comparison, making these methods unreliable. Nevertheless, small trials with favourable findings may be overrepresented, and relevant unpublished or non-English-language evidence may have been missed. These possibilities, together with small sample sizes and methodological limitations, warrant cautious interpretation of the apparent benefits and preclude strong clinical recommendations.

### 4.4. Implications for Practice, Policy, and Future Research

Despite heterogeneity across studies, the included RCTs generally showed favourable clinical and mycological outcomes with EO-based interventions, with no statistically significant differences detected against conventional antifungal therapies in small, underpowered trials without non-inferiority margins. However, the limited number of heterogeneous human studies and the inadequate and inconsistent assessment of safety and tolerability preclude their routine clinical implementation at present. Based on the findings of the present systematic review, further research into EO-based treatments for denture stomatitis is warranted. Standardised in vitro studies should first identify formulations with reproducible activity against both reference strains and clinical *Candida* isolates, including biofilms formed on denture-base acrylic. Only formulations demonstrating favourable and reproducible in vitro results should subsequently be evaluated in adequately powered RCTs, using standardised protocols and systematic assessment of safety, tolerability, and patient-reported outcomes.

## 5. Conclusions

This systematic review identified eight RCTs evaluating essential-oil-based or other plant-derived volatile interventions for oral candidiasis. All included trials were conducted in patients with denture stomatitis, so the conclusions apply to denture stomatitis only and cannot be extrapolated to other forms of oral candidiasis. Across the included studies, these interventions were associated with clinical improvement and reductions in *Candida* burden. In placebo-controlled trials, *Pelargonium graveolens* and *Satureja hortensis* gels produced superior clinical and mycological outcomes than placebo. In trials using conventional antifungal comparators, no statistically significant differences in clinical outcomes were detected in small, underpowered trials without non-inferiority margins, which does not demonstrate equivalence; mycological efficacy was less consistent, with nystatin or miconazole achieving greater reductions in fungal burden in some studies. These findings suggest that selected EO-based formulations may have therapeutic potential for denture stomatitis, particularly as topical interventions used alongside denture hygiene and disinfection measures. However, the current evidence is insufficient to support their use as substitutes for established antifungal therapies because of the small number of trials, small sample sizes, heterogeneity in formulations and treatment protocols, inconsistent outcome assessment, short follow-up periods, and sparse systematic assessment of safety and tolerability. This conclusion rests primarily on clinical and mycological outcomes; the safety data are insufficient to draw firm conclusions, and safety should not be assumed to have been adequately assessed. Future research should first identify chemically characterised EO-based formulations with reproducible anti-*Candida* activity in standardised in vitro models, including denture-base biofilms. Promising formulations should subsequently be evaluated in well-designed RCTs using standardised protocols, clinically relevant and consistently measured outcomes, longer follow-up, and systematic safety assessment.

## Figures and Tables

**Table 1:** Eligibility criteria for studies included in the systematic review.

Inclusion Criteria	Exclusion Criteria
Randomized controlled trials Clinically and mycologically diagnosed oral candidiasis, including denture-associated stomatitis Intervention: essential oil or other plant-derived volatile compound (incl. supercritical fluid extracts), applied topically. Presence of an adequate comparator: a conventional antifungal agent or placebo/vehicle Clinical and/or mycological outcomes reported Full text available, English language	Non-randomized designs (in vitro studies, case reports/series, single arm/open-label without control) Animal studies Population without confirmed oral candidiasis Intervention based on non-volatile plant preparations (extracts, tinctures, tannins, propolis, fixed oils) or combinations with other active herbal preparations outside the review’s scope, in which the effect of the eligible essential oil or volatile-containing preparation could not be isolated. Lack of an adequate comparator (e.g., herbal vs. herbal comparison, or two formulations of the same substance differing only in vehicle) Reviews, meta-analyses, conference abstracts, letters Trial registrations without a retrievable completed publication Non-English publications

**Table 3:** Characteristics of participants in the included studies.

Study and Reference	Sample Size Calculation	Participants, *n*	Female /Male, n	Age		Clinical Condition
				Mean	Range	
Amanlou et al. [25]	NR	24	14/10	60.83	45–83	Denture stomatitis; Newton types II/III
Araújo et al. [26]	Yes	36	27/9	57	40–70	Denture-related oral candidiasis; Newton types I/II/III
Catalán et al. [27]	NR	27	26/1	63.5 ± 7.44	50–77	Denture stomatitis; Newton type II
Gonoudi et al. [28]	NR	28	7/21	EG: 55.86 ± 10.04 CG: 60.93 ± 13.04	EG: 37–67 CG: 38–82	Denture stomatitis; Newton types II/III
Iyer et al. [29]	Yes	42	7/35	EG: 61.57 ± 7.8 CG: 62.86 ± 6.5	48–71	Denture stomatitis; Newton types I/II/III
Sabzghabaee et al. (*P. graveolens*) [30]	NR	80	51/29	61.39 ± 9.038	38–78	Denture stomatitis; Newton types II/III
Sabzghabaee et al. (*S. hortensis*) [31]	NR	80	49/31	62.91 ± 7.34	NR	Denture stomatitis; Newton types II/III
Sefidgar et al. [32]	NR	30	17/13	EG: 61.33 ± 9.49 CG: 58.13 ± 8.30	NR	Denture stomatitis; Newton types II/III

NR—not reported; EG—experimental group; CG—control group; *n*—number of participants.

**Table 4:** Characteristics of plant-derived volatile substances and preparations used in the included studies.

Study and Reference	Plant-Derived Volatile Substance	Plant Part Used	Extraction Method/Source	Chemical Composition Reported	Clinical Concentration	Pharmaceutical Formulation
Amanlou et al. [25]	*Zataria multiflora* EO	Leaves	Steam distillation	Thymol 49.5%; carvacrol 13.4%; p-cymene 12.3%; linalool 6.1%; γ-terpinene 4.1%	0.1%	Gel (base NR)
Araújo et al. [26]	*Cinnamomum zeylanicum* EO	Leaves	Hydrodistillation	Eugenol, main constituent	0.5 mg/mL	Mouthwash + denture spray (carboxymethylcellulose, propylene glycol, Tween 80)
Catalán et al. [27]	*Melaleuca alternifolia* EO	NR	Commercial EO	NR	1 mL/5 mL conditioner liquid	Coe-Comfort tissue conditioner
Gonoudi et al. [28]	*Zataria multiflora* EO	NR	Commercial EO (Gastroli^®^ Bridge Essence, Kashan, Iran)	Thymol + carvacrol	Unclear: 0.05% EO; also reported as 5 mg/mL	Oral solution (base NR)
Iyer et al. [29]	Clove/cinnamon SCF	Clove buds; cinnamon bark	Supercritical CO_2_ extraction	Eugenol; cinnamaldehyde; benzaldehyde	256 µg/mL clove + 32 µg/mL cinnamon	Emulgel (badam gum, Tween 80/PEG 400)
Sabzghabaee et al. (*P. graveolens*) [30]	*Pelargonium graveolens* EO	NR	Commercial oil (Barij^®^ Essence Company, Kashan, Iran)	β-citronellol; geraniol	1%	Gel (carboxymethylcellulose, Carbomer 934, Plastibase)
Sabzghabaee et al. (*S. hortensis*) [31]	*Satureja hortensis* EO	NR	Commercial oil (Barij^®^ Essence Company, Kashan, Iran)	Carvacrol; γ-terpinene; p-cymene	1%	Gel (carboxymethylcellulose, Carbomer 934, Plastibase)
Sefidgar et al. [32]	*Artemisia sieberi* EO	NR	NR	β-thujone, camphor, α-thujone reported in background literature	1%	Mouthwash (base NR)

EO—essential oil; NR—not reported; SCF—supercritical fluid extracts.

**Table 5:** Interventions and protocols in included RCTs.

Study	Experimental Intervention; n	Comparator; n	Treatment Regimen	Denture Management During Treatment
Amanlou et al. [25]	*Z. multiflora* EO 0.1% gel; n = 12	Miconazole 2% gel; n = 12	Topical, 4×/day, 14 days	Gel on denture base, 2.5 mL, 4×/day, usual denture-wearing and cleaning regimen
Araújo et al. [26]	*C. zeylanicum* EO 0.5 mg/mL mouthwash + spray; n = 18	Nystatin 100,000 IU/mL mouthwash + spray; n = 18	Rinse with 10 mL, 1 min, 3×/day, 15 days	Spray 3×/day, application for 1 min; denture brushing with soap; dry overnight storage
Catalán et al. [27]	*M. alternifolia* (1 mL) + Coe-Comfort tissue conditioner (4 mL); n = 9	Nystatin (2 mL) + Coe-Comfort (3 mL); n = 9; Coe-Comfort (5 mL); n = 9	Maxillary denture liner; 24 h/day wear; 12 days; reline: days 0, 4, 8	1-mm reduction in the denture intaglio surface before initial lining; 24 h/day wear
Gonoudi et al. [28]	*Z. multiflora* EO 0.05% solution; n = 14	Nystatin 100,000 IU/mL suspension; n = 14	Rinse with 5 mL, 2 min, 4×/day, 14 days	Denture use only during meals; post-meal brushing; overnight removal; water storage
Iyer et al. [29]	Clove/cinnamon emulgel; n = 21	Clotrimazole 1% gel; n = 21	Topical, 3×/day, 21 days	NR
Sabzghabaee et al. (*P. graveolens*) [30]	*P. graveolens* EO 1% gel; n = 40	Placebo gel; n = 40	Topical, 2×/day, 14 days	Gel on denture base, 2×/day; overnight removal; hygiene instructions
Sabzghabaee et al. (*S. hortensis*) [31]	*S. hortensis* EO 1% gel; n = 40	Placebo gel; n = 40	Topical, 2×/day, 14 days	Gel on denture base, 2×/day; hygiene instructions
Sefidgar et al. [32]	*A. sieberi* 1% mouthwash; n = 15	Nystatin 500,000 Units mouthwash; n = 15	Rinse with 20 mL, 1 min, 4×/day; till clinical resolution	Denture use discontinued

EO—essential oil; n—number of participants in the studied group; IU—international units; mL—millilitres; NR—not reported; h—hours; min—minutes; ×/day—times per day.

**Table 6:** Clinical and mycological outcome measures and main findings of the included randomised controlled trials.

Study	Clinical Outcome Measures	Mycological Outcome Measures	Assessment Time Points and Follow-Up	Main Clinical Findings	Main Mycological Findings
Amanlou et al. [25]	Palatal erythema surface (cm^2^).	*Candida* spp. colony counts from palatal mucosa and maxillary denture surface.	Baseline; days 7, 14 during treatment; days 21 and 28 (post-treatment follow-up).	Significant reduction in palatal erythema in both groups; no significant between-group differences at any assessment.	Significant reduction in *Candida* spp. counts on palatal mucosa in both groups; miconazole more effective in reducing denture-surface colony counts.
Araújo et al. [26]	Clinical signs according to the Newton classification.	*Candida* spp. CFU from oral mucosa and dentures.	Baseline and day 16 (24 h after completing 15-day treatment); no further follow-up.	Improvement of clinical signs in both groups; between-group comparison of clinical change insignificant (*p* = 0.4124).	Reduction in *Candida* spp. counts in both groups; greater reductions with nystatin than *C. zeylanicum* on mucosa (89% vs. 61%) and denture surfaces (83% vs. 33%); *C. tropicalis* eliminated in the EO group at both sites.
Catalán et al. [27]	Palatal inflammation score (0 = none, 1 = slight, 2 = moderate, 3 = severe).	*C. albicans* CFU from denture-bearing palatal mucosa, reported as mean log10 CFU.	Baseline; days 4 and 8; day 12 (end of treatment); no further follow-up.	Complete clinical resolution in 8/9 patients in both active-treatment groups at day 12; both superior to Coe-Comfort alone (p=0.001); no difference between EO group and nystatin (p=0.403).	Significant reduction in *C. albicans* growth with both active treatments versus Coe-Comfort alone; no difference between active treatments at day 12 (p=0.77).
Gonoudi et al. [28]	Palatal erythema surface (mm^2^).	*C. albicans* CFU from palatal swabs.	Baseline and day 14 (end of treatment); no further follow-up.	Significant reduction in palatal erythema in both groups; no between-group difference (p=0.256)	Significant reduction in *C. albicans* CFUs from baseline in both groups; no between-group difference (p=0.593).
Iyer et al. [29]	Palatal erythema according to the Newton classification.	*Candida* spp. CFU/mL from palatal mucosa and denture surfaces.	Baseline; days 7, 14, and 21 during treatment; no further follow-up.	Significant improvement in clinical signs in both groups; greater improvement and numerically higher cure rate with clove/cinnamon emulgel at day 21 (47.6% vs. 42.9%; 10/21 vs. 9/21).	Significant reduction in *Candida* spp. CFUs over time in both groups; greater reduction on denture surfaces with the emulgel.
Sabzghabaee et al. (*P. graveolens*) [30]	Palatal erythema; complete, partial, or no clinical recovery.	*Candida* spp. colony counts from palatal mucosa and denture fitting surface.	Baseline; days 7, 14 (end of treatment); no further follow-up.	Significantly higher recovery rates with EO gel than placebo: complete recovery, 34% vs. 5%; partial recovery, 56% vs. 25%.	Significantly greater reduction in fungal colony counts with EO gel than placebo.
Sabzghabaee et al. (*S. hortensis*) [31]	Palatal erythema; complete, partial, or no clinical recovery.	*C. albicans* colony counts from palatal mucosa.	Baseline; day 14 (end of treatment); day 21 (post-treatment follow-up).	Higher recovery rates with EO gel than placebo: complete recovery, 30% vs. 5%; partial recovery, 52.5% vs. 25%.	Significantly greater reduction in *C. albicans* colony counts with EO gel than placebo.
Sefidgar et al. [32]	Clinical resolution of denture stomatitis.	Semiquantitative *Candida* spp. Colony growth score from palatal lesion swabs.	Baseline; weekly until clinical resolution (max. 2 weeks); week 3 follow-up.	Identical clinical resolution in both groups: 14/15 patients per group after 1 week and the remaining patient after 2 weeks.	Significant within-group reduction in colony-growth scores (p<0.0001); no between-group difference (p>0.05).

CFU—colony-forming units; EO—essential oil; NR—not reported; mL—millilitres. p—p values are reported as provided in the original studies.

## Data Availability

RoB 2 judgements supporting this review are provided in the Supplementary Materials (Table S3).

## Supplementary Materials

The following supporting information can be downloaded at: https://www.mdpi.com/article/10.3390/jcm15218617/s1, Table S1: Full electronic search strategies across databases-Full electronic search strategies for PubMed, Embase, Scopus, and Cochrane CENTRAL; Table S2: Detailed line-by-line search strategy for the Cochrane Library (CENTRAL); Table S3: Risk of bias assessment supporting information; Table S4: Safety, tolerability, and patient-reported outcomes in the included trials; PRISMA 2020 Checklist; Figure S1: Risk-of-bias assessment using the RoB 2 tool for the secondary mycological outcome at the end of treatment.
