# Supplementary material for: Essential Oils and Plant-Derived Volatile Compounds in the Treatment of Oral Candidiasis: A Systematic Review of Randomized Controlled Trials in Denture Stomatitis
Source: J Clin Med. 2026 Nov 5;15(21):8617. doi: 10.3390/jcm15218617 (PMC13764656; doi:10.3390/jcm15218617)
Supplement: Supplementary file 1 [file jcm-15-08617-s001.zip › Supplementary_Figure_S1_Risk_of_Bias_Mycological_Outcome.pdf]

Author (year)	D1	D2	D3	D4	D5	Overall
Amanlou (2006)	+	+	+	+	!	!
Araujo (2021)	+	+	+	+	+	+
Catalan (2008)	!	+	+	+	!	!
Gonoudi (2021)	+	+	+	+	+	+
Iyer (2022)	+	+	+	+	+	+
Sabzghabaee (2011)	+	+	+	+	!	!
Sabzghabaee (2012)	+	+	+	+	!	!
Sefidgar (2010)	!	+	+	+	!	!

Legend

+	Low risk
!	Some concerns
-	High risk
**D1**	Randomisation process
**D2**	Deviations from the intended interventions
**D3**	Missing outcome data
**D4**	Measurement of the outcome
**D5**	Selection of the reported result

Figure S1. Risk-of-bias assessment using the RoB 2 tool for the secondary mycological outcome at the end of treatment. D1, randomisation process; D2, deviations from the intended interventions; D3, missing outcome data; D4, measurement of the outcome; D5, selection of the reported result (the risk that the reported result was chosen from several possible measurements or analyses).
