# Supplementary material for: Essential Oils and Plant-Derived Volatile Compounds in the Treatment of Oral Candidiasis: A Systematic Review of Randomized Controlled Trials in Denture Stomatitis
Source: J Clin Med. 2026 Nov 5;15(21):8617. doi: 10.3390/jcm15218617 (PMC13764656; doi:10.3390/jcm15218617)
Supplement: Supplementary file 1 [file jcm-15-08617-s001.zip › Supplementary_Tables_S1_S2_Search_Strategies.pdf]

Supplementary Materials - Electronic Search Strategies

Table S1. Full electronic search strategies across databases

Databases searched: PubMed, Embase, Scopus, and Cochrane Central Register of Controlled Trials (CENTRAL), via Cochrane Library.

Search date: 20 July 2026

Database	Search string	Records retrieved
**PubMed**	("oral candidiasis"[Title/Abstract] OR "oral candidiases"[Title/Abstract] OR "oral candidosis"[Title/Abstract] OR "oral candidoses"[Title/Abstract] OR "candidiasis, oral"[MeSH Terms] OR "oropharyngeal candidiasis"[Title/Abstract] OR "denture stomatitis"[Title/Abstract] OR "denture-related stomatitis"[Title/Abstract] OR "candida-associated denture stomatitis"[Title/Abstract] OR "stomatitis, denture"[MeSH Terms] OR "oral moniliasis"[Title/Abstract] OR "oral moniliases"[Title/Abstract] OR "oral thrush"[Title/Abstract] OR "thrush"[Title/Abstract]) AND ("oils, volatile"[MeSH Terms] OR "essential oil*"[Title/Abstract] OR "volatile oil*"[Title/Abstract] OR "volatile compound*"[Title/Abstract] OR "plant extract*"[Title/Abstract] OR "medicinal plant*"[Title/Abstract] OR "supercritical fluid extract*"[Title/Abstract] OR "melaleuca"[Title/Abstract] OR "tea tree*"[Title/Abstract] OR "zataria multiflora"[Title/Abstract] OR "pelargonium"[Title/Abstract] OR "geranium"[Title/Abstract] OR "satureja"[Title/Abstract] OR "cinnamon*"[Title/Abstract] OR "cinnamomum*"[Title/Abstract] OR "clove*"[Title/Abstract] OR "syzygium aromaticum"[Title/Abstract] OR "origanum*"[Title/Abstract] OR "thyme*"[Title/Abstract] OR "thymol"[Title/Abstract] OR "carvacrol"[Title/Abstract] OR "eugenol"[Title/Abstract])	159
**Embase**	('mouth candidiasis'/exp OR 'oral candidiasis':ti,ab,kw OR 'oral candidiases':ti,ab,kw OR 'oral candidosis':ti,ab,kw OR 'oral candidoses':ti,ab,kw OR 'oropharyngeal candidiasis':ti,ab,kw OR 'denture stomatitis':ti,ab,kw OR 'denture-related stomatitis':ti,ab,kw OR 'candida-associated denture stomatitis':ti,ab,kw OR 'oral moniliasis':ti,ab,kw OR 'oral moniliases':ti,ab,kw OR 'oral thrush':ti,ab,kw OR 'thrush':ti,ab,kw) AND ('essential oil'/exp OR 'essential oil*':ti,ab,kw OR 'volatile oil*':ti,ab,kw OR 'volatile compound*':ti,ab,kw OR 'plant extract*':ti,ab,kw OR 'medicinal plant*':ti,ab,kw OR 'supercritical fluid extract*':ti,ab,kw OR 'melaleuca':ti,ab,kw OR 'tea tree*':ti,ab,kw OR 'zataria multiflora':ti,ab,kw OR 'pelargonium':ti,ab,kw OR 'geranium':ti,ab,kw OR 'satureja':ti,ab,kw OR 'cinnamon*':ti,ab,kw OR 'cinnamomum*':ti,ab,kw OR 'clove*':ti,ab,kw OR 'syzygium aromaticum':ti,ab,kw OR 'origanum*':ti,ab,kw OR 'thyme*':ti,ab,kw OR 'thymol':ti,ab,kw OR 'carvacrol':ti,ab,kw OR 'eugenol':ti,ab,kw)	278
**Scopus**	TITLE-ABS-KEY("oral candidiasis" OR "oral candidiases" OR "oral candidosis" OR "oral candidoses" OR "oropharyngeal candidiasis" OR "denture stomatitis" OR "denture-related stomatitis" OR "candida-associated denture stomatitis" OR "oral moniliasis" OR "oral moniliases" OR "oral thrush" OR "thrush") AND TITLE-ABS-KEY("essential oil*" OR "volatile oil*" OR "volatile compound*" OR "plant extract*" OR "medicinal plant*" OR "supercritical fluid extract*" OR "melaleuca" OR "tea tree*" OR "zataria multiflora" OR "pelargonium" OR "geranium" OR "satureja" OR "cinnamon*" OR "cinnamomum*" OR "clove*" OR "syzygium aromaticum" OR "origanum*" OR "thyme*" OR "thymol" OR "carvacrol" OR "eugenol")	457
**Cochrane Library (CENTRAL)**	(("oral candidiasis" OR "oral candidiases" OR "oral candidosis" OR "oral candidoses" OR "oropharyngeal candidiasis" OR "denture stomatitis" OR "denture-related stomatitis" OR "candida-associated denture stomatitis" OR "oral moniliasis" OR "oral moniliases" OR "oral thrush" OR "thrush"):ti,ab,kw OR [mh Candidiasis, Oral] OR [mh Stomatitis, Denture]) AND (((essential NEXT oil*) OR (volatile NEXT oil*) OR (volatile NEXT compound*) OR (plant NEXT extract*) OR (medicinal NEXT plant*) OR (supercritical NEXT fluid NEXT extract*) OR melaleuca OR (tea NEXT tree*) OR "zataria multiflora" OR pelargonium OR	25

Database	Search string	Records retrieved
	geranium OR satureja OR cinnamon* OR cinnamomum* OR clove* OR "syzygium aromaticum" OR origanum* OR thyme* OR thymol OR carvacrol OR eugenol):ti,ab,kw OR [mh Oils, Volatile])	

Table S2. Detailed line-by-line search strategy for the Cochrane Library (CENTRAL)

Search date: 20 July 2026

Line	Search terms	Results
**#1**	("oral candidiasis" OR "oral candidiases" OR "oral candidosis" OR "oral candidoses" OR "oropharyngeal candidiasis" OR "denture stomatitis" OR "denture-related stomatitis" OR "candida-associated denture stomatitis" OR "oral moniliasis" OR "oral moniliases" OR "oral thrush" OR "thrush"):ti,ab,kw	1,109
**#2**	((essential NEXT oil*) OR (volatile NEXT oil*) OR (volatile NEXT compound*) OR (plant NEXT extract*) OR (medicinal NEXT plant*) OR (supercritical NEXT fluid NEXT extract*) OR melaleuca OR (tea NEXT tree*) OR "zataria multiflora" OR pelargonium OR geranium OR satureja OR cinnamon* OR cinnamomum* OR clove* OR "syzygium aromaticum" OR origanum* OR thyme* OR thymol OR carvacrol OR eugenol):ti,ab,kw	12,524
**#3**	MeSH descriptor: [Candidiasis, Oral] explode all trees	269
**#4**	MeSH descriptor: [Stomatitis, Denture] explode all trees	105
**#5**	MeSH descriptor: [Oils, Volatile] explode all trees	682
**#6**	#1 OR #3 OR #4	1,209
**#7**	#2 OR #5	12,681
**#8**	#6 AND #7	25

Note: The final CENTRAL search set was #8 (#6 AND #7), yielding 25 records, consistent with Table S1. Set #6 combined the condition terms (#1 OR #3 OR #4), and set #7 combined the intervention terms (#2 OR #5). Counts for individual sets are not additive because records may occur in more than one set; OR retrieves the union of sets, whereas AND retrieves their intersection. Counts are those reported for the original search and have not been updated by rerunning the search.

*Abbreviations: ti,ab,kw = title, abstract, keyword fields; NEXT = Cochrane proximity operator requiring adjacent terms in the specified order; * = truncation (wildcard) operator; MeSH = Medical Subject Headings; explode all trees = includes the specified MeSH term and all of its narrower (more specific) subject headings; /exp = Embase Emtree explode function; TITLE-ABS-KEY = Scopus field code searching title, abstract, and author keywords.*
