# Supplementary material for: Essential Oils and Plant-Derived Volatile Compounds in the Treatment of Oral Candidiasis: A Systematic Review of Randomized Controlled Trials in Denture Stomatitis
Source: J Clin Med. 2026 Nov 5;15(21):8617. doi: 10.3390/jcm15218617 (PMC13764656; doi:10.3390/jcm15218617)
Supplement: Supplementary file 1 [file jcm-15-08617-s001.zip › Supplementary_Table_S3_RoB2_Supporting_Information.pdf]

Table S3 Risk of bias assessment supporting information

Outcome-specific RoB 2 signalling-question responses and assessor notes for the eight included randomized trials. The assessments address the effect of assignment to intervention. Clinical and mycological outcomes are presented separately, with the exact comparison and endpoint specified for each assessment.

The tables present the final assessor judgements, signalling-question responses and supporting explanations for each domain. Supporting explanations summarize the evidence and reasoning recorded during assessment; they are not necessarily verbatim quotations. Source page and table references are provided where available.

Response codes: Y = Yes; PY = Probably yes; PN = Probably no; N = No; NI = No information; NA = Not applicable. Question identifiers refer to the RoB 2 tool for individually randomized parallel-group trials. The question key on the next page provides abbreviated labels, not replacement wording for the original tool.

Overview of assessor judgements

Study	Outcome	D1	D2	D3	D4	D5	Overall
Amanlou (2006)	Clinical	L	L	L	L	SC	SC
Araujo (2021)	Clinical	L	L	L	L	L	L
Catalan (2008)	Clinical	SC	L	L	SC	SC	SC
Gonoudi (2021)	Clinical	L	L	L	L	L	L
Iyer (2022)	Clinical	L	L	L	L	L	L
Sabzghabaee (2011)	Clinical	L	L	L	L	SC	SC
Sabzghabaee (2012)	Clinical	L	L	L	L	SC	SC
Sefidgar (2010)	Clinical	SC	L	L	L	SC	SC
Amanlou (2006)	Mycological	L	L	L	L	SC	SC
Araujo (2021)	Mycological	L	L	L	L	L	L
Catalan (2008)	Mycological	SC	L	L	L	SC	SC
Gonoudi (2021)	Mycological	L	L	L	L	L	L
Iyer (2022)	Mycological	L	L	L	L	L	L
Sabzghabaee (2011)	Mycological	L	L	L	L	SC	SC
Sabzghabaee (2012)	Mycological	L	L	L	L	SC	SC
Sefidgar (2010)	Mycological	SC	L	L	L	SC	SC

L = Low risk; SC = Some concerns. D1–D5 correspond to the five domains listed in the question key. Overall judgements are the final assessor judgements.

Assessment tool: Cochrane Risk of Bias 2 for individually randomized parallel-group trials, effect of assignment to intervention. Reference: Sterne JAC et al. RoB 2: a revised tool for assessing risk of bias in randomised trials. BMJ. 2019;366:l4898. doi:10.1136/bmj.l4898.

Signalling question key

Domain	Item	Abbreviated question topic
D1	1.1	Random allocation sequence
D1	1.2	Concealment until enrolment and assignment
D1	1.3	Baseline differences suggesting a randomization problem
D2	2.1	Participants aware of assigned intervention
D2	2.2	Carers and intervention providers aware of assignment
D2	2.3	Deviations arising from the experimental context
D2	2.4	Deviations likely to affect the outcome
D2	2.5	Deviations balanced between groups
D2	2.6	Appropriate analysis of assignment effect
D2	2.7	Potential substantial impact of failure to analyse as randomized
D3	3.1	Outcome data available for all or nearly all participants
D3	3.2	Evidence that missing data did not bias the result
D3	3.3	Missingness could depend on the true outcome value
D3	3.4	Missingness likely to depend on the true outcome value
D4	4.1	Inappropriate outcome measurement method
D4	4.2	Measurement or ascertainment could differ between groups
D4	4.3	Outcome assessors aware of assigned intervention
D4	4.4	Assessment could be influenced by knowledge of intervention
D4	4.5	Assessment likely influenced by knowledge of intervention
D5	5.1	Analysis followed a plan finalized before unblinded data were available
D5	5.2	Selection based on results from multiple eligible outcome measurements
D5	5.3	Selection based on results from multiple eligible analyses

Domains: D1 Randomization process; D2 Deviations from intended interventions; D3 Missing outcome data; D4 Measurement of the outcome; D5 Selection of the reported result.

Amanlou (2006) clinical outcome

Domains 1 to 3

Intervention: Zataria multiflora 0.1% essential oil gel, 4 times daily for 14 days

Comparator: Miconazole 2% gel, 4 times daily for 14 days

Outcome assessed: Clinical outcome: erythema surface of palatal mucosa at day 14

Result assessed: Mean erythema score at day 14; Figure 1

Evidence sources recorded: Journal article(s)

D1 Randomization process

Signalling-question responses	Assessor judgement
1.1: PY; 1.2: PY; 1.3: N	Low

Note for 1.1&1.2: Patients were reported to be randomly assigned to the miconazole or Z. multiflora gel group; however, the method used to generate the random allocation sequence was not described.

Note for 1.3: The groups were reported to be similar at baseline with respect to age, sex, systemic disease history, denture-wearing characteristics, Candida colony counts and erythema surface. No baseline differences suggesting a problem with the randomization process were reported.

D2 Deviations from intended interventions

Signalling-question responses	Assessor judgement
2.1: N; 2.2: N; 2.3: NA; 2.4: NA; 2.5: NA; 2.6: Y; 2.7: NA	Low

Note for 2.1&2.2: Double-blind; matched gels (p. 967). Double-blind study; treatment codes were not known (p. 967).

Note for 2.6: All 24 randomized participants were reported in two groups of 12; no exclusions from analysis were reported.

D3 Missing outcome data

Signalling-question responses	Assessor judgement
3.1: Y; 3.2: NA; 3.3: NA; 3.4: NA	Low

Note for 3.1: No missing outcome data or exclusions were reported.

Amanlou (2006) clinical outcome

Domains 4 and 5

Outcome assessed: Clinical outcome: erythema surface of palatal mucosa at day 14

D4 Measurement of the outcome

Signalling-question responses	Assessor judgement
4.1: N; 4.2: N; 4.3: N; 4.4: NA; 4.5: NA	Low

Note for 4.1: Erythema surface was assessed clinically using a predefined six-point grading scale; the measurement method was appropriate for this outcome.

Note for 4.2: The same clinical assessment procedure was used for both intervention groups, and all participants were examined by one dentist.

Note for 4.3: The study was reported as double-blind, and treatment codes were not known during the clinical examination.

D5 Selection of the reported result

Signalling-question responses	Assessor judgement
5.1: NI; 5.2: N; 5.3: N	Some concerns

Note for 5.1: No pre-specified analysis plan, protocol, statistical analysis plan, or trial registration record was available to confirm that the analysis was finalized before outcome data were examined.

Note for 5.2: Erythema was assessed at several post-baseline time points; however, results for all reported follow-up visits were presented. There was no indication that the day 14 result was selected on the basis of its finding.

Note for 5.3: The report suggests a single standard between-group analysis for the outcome. No multiple adjusted, subgroup, or alternative analyses were reported; however, the analysis details were limited.

Overall judgement

Some concerns

Araujo (2021) clinical outcome

Domains 1 to 3

Intervention: Cinnamomum zeylanicum essential oil 0.5 mg/mL mouthwash and denture spray, 3 times daily for 15 days

Comparator: Nystatin 100,000 IU/mL, 3 times daily for 15 days

Outcome assessed: Clinical outcome: Newton classification of denture stomatitis at day 16

Result assessed: Newton classification at day 16; Table 3

Evidence sources recorded: Journal article(s); Non-commercial trial registry record (e.g. ClinicalTrials.gov record)

D1 Randomization process

Signalling-question responses	Assessor judgement
1.1: Y; 1.2: Y; 1.3: N	Low

Note for 1.1&1.2: The participants were randomly allocated to treatment and control groups. The allocation sequence was generated with a computer program (random allocation). Allocation concealment was ensured by an individual who was not one of the study investigators to avoid selection bias and the influence of the investigators on the intervention.

Note for 1.3: The groups were reported to be homogeneous at baseline with respect to clinical, mycological and denture-use characteristics

D2 Deviations from intended interventions

Signalling-question responses	Assessor judgement
2.1: N; 2.2: N; 2.3: NA; 2.4: NA; 2.5: NA; 2.6: Y; 2.7: NA	Low

Note for 2.1&2.2: Blinding: For double-blinding purposes, the tested substances (C. zeylanicum and nystatin) had the same color, taste, and odor and were stored in identical dark vials labeled A or B. As a result, neither the participants nor the investigators were aware of the allocation groups. A single investigator and the pharmacist responsible for the formulations were aware of the composition of the products. The investigator responsible for randomization, and the one responsible for preparing the products did not participate in the clinical stage of the study

Note for 2.6: Thirty-six participants were randomized equally between the two groups and analysed as allocated, with 18 participants in each group.

D3 Missing outcome data

Signalling-question responses	Assessor judgement
3.1: Y; 3.2: NA; 3.3: NA; 3.4: NA	Low

Note for 3.1: Clinical outcome data were reported for all 36 randomized participants, with 18 participants in each treatment group at post-treatment assessment

Araujo (2021) clinical outcome

Domains 4 and 5

Outcome assessed: Clinical outcome: Newton classification of denture stomatitis at day 16

D4 Measurement of the outcome

Signalling-question responses	Assessor judgement
4.1: N; 4.2: N; 4.3: N; 4.4: NA; 4.5: NA	Low

Note for 4.1: Clinical denture stomatitis was assessed using the Newton classification by a calibrated examiner; intraexaminer agreement was substantial (Kappa = 0.822).

Note for 4.2: The same clinical examination and Newton classification were used for both intervention groups at baseline and post-treatment assessment.

Note for 4.3: The clinical investigators were reported to be blinded to treatment allocation throughout the clinical stage of the study.

D5 Selection of the reported result

Signalling-question responses	Assessor judgement
5.1: PY; 5.2: N; 5.3: N	Low

Note for 5.1: A registered trial record was available and specified clinical improvement after the 15-day intervention as the primary outcome. The reported clinical outcome was consistent with the registered study objective.

Note for 5.2: Clinical assessment was performed at baseline and once after the 15-day intervention. There was no indication that the day 16 result was selected from multiple post-treatment time points on the basis of its findings.

Note for 5.3: The report describes a standard Mann–Whitney analysis of the Newton classification. No multiple adjusted, subgroup or alternative analyses of the clinical outcome were reported.

Overall judgement

Low

Catalan (2008) clinical outcome

Domains 1 to 3

Intervention: Melaleuca alternifolia oil mixed with Coe-Comfort tissue conditioner, applied as a denture reline and replaced every 4 days for 12 days

Comparator: Nystatin mixed with Coe-Comfort tissue conditioner, applied as a denture reline and replaced every 4 days for 12 days

Outcome assessed: Clinical outcomes: remission of denture stomatitis normal palatal mucosa at day 12

Result assessed: Normal palatal mucosa at day 12: 8/9 participants in the Melaleuca alternifolia group versus 8/9 participants in the nystatin group.

Evidence sources recorded: Journal article(s)

D1 Randomization process

Signalling-question responses	Assessor judgement
1.1: PY; 1.2: NI; 1.3: PN	Some concerns

Note for 1.1&1.2: The report states that patients were randomly divided into three 9-person treatment groups, but it does not describe how the random allocation sequence was generated. The report provides no information on allocation concealment.

Note for 1.3: Baseline characteristics were described for the full study sample, but baseline data were not reported separately for the three randomized groups.

D2 Deviations from intended interventions

Signalling-question responses	Assessor judgement
2.1: NI; 2.2: NI; 2.3: PN; 2.4: NA; 2.5: NA; 2.6: Y; 2.7: NA	Low

Note for 2.1&2.2: The report does not state whether participants were blinded to the intervention received. The report does not state whether the clinicians preparing and applying the tissue conditioners were blinded to treatment allocation.

Note for 2.3: Participants attended scheduled sessions every 4 days, when the previous tissue conditioner was removed and a new liner containing the allocated treatment was applied. No treatment discontinuations, crossovers, additional antifungal treatment, or other deviations from the intended intervention arising from the experimental context were reported. Such deviations probably did not occur.

Note for 2.6: Clinical outcome data at day 12 were reported for all randomized participants: 9 participants in each of the three groups. No exclusions or losses to follow-up were reported.

D3 Missing outcome data

Signalling-question responses	Assessor judgement
3.1: Y; 3.2: NA; 3.3: NA; 3.4: NA	Low

Note for 3.1: Clinical outcome data at day 12 were reported for all participants in the two compared groups: 8 of 9 participants in the Melaleuca alternifolia group and 8 of 9 participants in the nystatin group had normal palatal mucosa. The report did not describe any losses to follow-up.

Catalan (2008) clinical outcome

Domains 4 and 5

Outcome assessed: Clinical outcomes: remission of denture stomatitis normal palatal mucosa at day 12

D4 Measurement of the outcome

Signalling-question responses	Assessor judgement
4.1: N; 4.2: N; 4.3: NI; 4.4: PY; 4.5: PN	Some concerns

Note for 4.1: Palatal inflammation was assessed clinically using a predefined four-category erythema scale: no, slight, moderate or severe inflammation. This was an appropriate method for evaluating clinical remission of denture stomatitis

Note for 4.2: Palatal mucosa was assessed at each visit using the same clinical erythema scale by one clinician for all treatment groups.

Note for 4.3: The report does not state whether the clinician who assessed palatal inflammation was blinded to treatment allocation.

Note for 4.4&4.5: The clinician graded visible palatal inflammation on an ordinal scale, which requires judgement and could be influenced by knowledge of allocation. Assessor blinding was not reported. The same clinician and assessment procedure were used across groups, and no evidence that ratings were actually influenced was identified. This supports some concerns, rather than assuming that an unreported blinding procedure was present (pp. 329–330).

D5 Selection of the reported result

Signalling-question responses	Assessor judgement
5.1: NI; 5.2: N; 5.3: N	Some concerns

Note for 5.1: No trial protocol, prospective trial registration record or statistical analysis plan was available. Therefore, it was not possible to determine whether the analysis of clinical remission at day 12 followed a pre-specified analysis plan finalized before unblinded outcome data were available

Note for 5.2: Clinical remission was reported at day 12, which was the final scheduled examination at the end of the 12-day treatment period. There was no indication that this end-of-treatment result was selected from multiple eligible post-treatment time points on the basis of its findings.

Note for 5.3: The report described one clinical analysis using Friedman's test. There was no indication that the reported day 12 clinical result was selected from multiple alternative analyses on the basis of the findings.alyses.

Overall judgement

Some concerns

Gonoudi (2021) clinical outcome

Domains 1 to 3

Intervention: Zataria multiflora essential oil 0.05%, 5 mL mouth rinse for 2 minutes, four times daily for 14 days

Comparator: Nystatin suspension 100,000 units, 40 drops mouth rinse for 2 minutes, four times daily for 14 days

Outcome assessed: Clinical outcome: palatal erythema area (mm^2) at day 14

Result assessed: Mean palatal erythema area at day 14: 42.86 ± 55.39 mm^2 in the Zataria multiflora group versus 21.07 ± 34.42 mm^2 in the nystatin group; between-group P = 0.256.

Evidence sources recorded: Journal article(s)

D1 Randomization process

Signalling-question responses	Assessor judgement
1.1: Y; 1.2: Y; 1.3: N	Low

Note for 1.1&1.2: Stratified block randomization was used according to denture stomatitis type II or III. Allocation was based on computer-generated random numbers. Medications were dispensed by a person who was not involved in the study, according to the computer-generated random allocation sequence.

Note for 1.3: The groups were balanced for denture stomatitis type, with seven participants with type II and seven with type III stomatitis in each group. Baseline demographic and clinical characteristics showed no statistically significant between-group differences.

D2 Deviations from intended interventions

Signalling-question responses	Assessor judgement
2.1: NI; 2.2: PN; 2.3: PN; 2.4: NA; 2.5: NA; 2.6: Y; 2.7: NA	Low

Note for 2.1&2.2: The study was described as single-blind, but the report did not specify whether participants were blinded to the intervention received. Study medications were dispensed by another person according to the computer-generated random allocation sequence. This person had to know the assigned intervention in order to dispense either Zataria multiflora or nystatin.

Note for 2.3: Both groups received standardized instructions for medication use and denture hygiene. No protocol deviations, treatment switching or relevant co-interventions were reported; deviations arising because of the trial context were probably absent.

Note for 2.6: All 28 randomized participants were included in the two study groups, with 14 participants per group. The study reported between-group analysis of erythema using repeated-measures ANOVA.

D3 Missing outcome data

Signalling-question responses	Assessor judgement
3.1: Y; 3.2: NA; 3.3: NA; 3.4: NA	Low

Note for 3.1: Palatal erythema data before and after treatment were reported for all 28 randomized participants, with 14 participants in the Zataria multiflora group and 14 participants in the nystatin group. No losses to follow-up or exclusions from the outcome analysis were reported.

Gonoudi (2021) clinical outcome

Domains 4 and 5

Outcome assessed: Clinical outcome: palatal erythema area (mm²) at day 14

D4 Measurement of the outcome

Signalling-question responses	Assessor judgement
4.1: N; 4.2: N; 4.3: NI; 4.4: PN; 4.5: NA	Low

Note for 4.1: The erythematous area of the palate was outlined and measured in square millimetres using a transparent 5-mm squared grid. This was an appropriate method for measuring the extent of palatal erythema.

Note for 4.2: The same outline-and-grid measurement procedure was used at baseline and day 14 for participants in both intervention groups.

Note for 4.3: The report describes the trial as single-blind and states that medication was dispensed by a person not otherwise involved in the study. It does not identify the blinded party or establish that the erythema assessor was blinded. The response is therefore NI, not evidence of confirmed assessor blinding (pp. 61–62).

Note for 4.4&4.5: The affected area was outlined with a copying pencil and measured with a 5-mm squared transparent grid, with area reported in square millimetres (p. 62). The retained PN response reflects the assessor judgement that this structured quantitative procedure limited susceptibility to allocation knowledge. However, delineating the erythema boundary still requires clinical judgement, and the report does not establish assessor blinding. Thus, the low-risk judgement rests on an inference about measurement susceptibility, not on documented blinding.

D5 Selection of the reported result

Signalling-question responses	Assessor judgement
5.1: PY; 5.2: N; 5.3: N	Low

Note for 5.1: The report states that the trial was registered in the Iranian Registry of Clinical Trials in 2017. Palatal erythema was specified as an outcome and was assessed at baseline and after the planned 14-day treatment period. It is probably that the reported analysis followed a pre-specified plan.

Note for 5.2: Palatal erythema was measured at baseline and at one planned post-treatment assessment on day 14. There was no indication that the reported day 14 result was selected from multiple eligible post-treatment time points on the basis of its findings.

Note for 5.3: The report described repeated-measures ANOVA for comparison of erythema over time and between groups. There was no indication that the reported result was selected from multiple alternative analyses on the basis of the findings.

Overall judgement

Low

Iyer (2022) clinical outcome

Domains 1 to 3

Intervention: Optimized clove/cinnamon supercritical fluid extracts-loaded emulgel

Comparator: Commercially available antifungal gel (Candid® gel)

Outcome assessed: Clinical outcome: Newton's classification of denture stomatitis at day 21

Result assessed: At day 21, complete cure was observed in 10/21 participants in the clove/cinnamon emulgel group and 9/21 participants in the Candid® gel group. The remaining participants were classified as Newton stage I: 11/21 versus 7/21; Newton stage II: 0/21 versus 5/21; Newton stage III: 0/21 versus 0/21.

Evidence sources recorded: Journal article(s)

D1 Randomization process

Signalling-question responses	Assessor judgement
1.1: Y; 1.2: Y; 1.3: N	Low

Note for 1.1&1.2: The supplementary materials state that randomization was performed using computer-generated random numbers. The supplementary CONSORT chart states that both the investigator and study participants were blinded. Medication tubes were coded and distributed according to participant number and group allocation, supporting concealment of allocation during enrolment and assignment.

Note for 1.3: The groups had similar mean age and gender distribution. Baseline Newton's classification was broadly comparable: stage I, II and III were present in 6, 13 and 2 participants in the test group and 9, 10 and 2 participants in the control group, respectively. These differences do not suggest a problem with randomization.

D2 Deviations from intended interventions

Signalling-question responses	Assessor judgement
2.1: N; 2.2: N; 2.3: NA; 2.4: NA; 2.5: NA; 2.6: Y; 2.7: NA	Low

Note for 2.1&2.2: The supplementary CONSORT chart states that study participants were blinded. Medication tubes were coded, so participants were not aware of the assigned intervention. The supplementary CONSORT chart states that the investigator was blinded. Study medications were supplied in coded tubes, and participants applied the assigned gel themselves according to the same written and verbal protocol.

Note for 2.6: Clinical response data at day 21 were reported for all 42 randomized participants: 21 participants in the clove/cinnamon emulgel group and 21 participants in the Candid® gel group.

D3 Missing outcome data

Signalling-question responses	Assessor judgement
3.1: Y; 3.2: NA; 3.3: NA; 3.4: NA	Low

Note for 3.1: Clinical response data at day 21 were reported for all 42 randomized participants. Newton's classification categories and complete cure status accounted for 21 participants in each intervention group, with no missing outcome data or exclusions reported.

Iyer (2022) clinical outcome

Domains 4 and 5

Outcome assessed: Clinical outcome: Newton's classification of denture stomatitis at day 21

D4 Measurement of the outcome

Signalling-question responses	Assessor judgement
4.1: N; 4.2: N; 4.3: N; 4.4: NA; 4.5: NA	Low

Note for 4.1: Denture stomatitis was assessed using Newton's classification: type I localized petechiae, type II diffuse erythema and type III erythema with papillary hyperplasia. This was an appropriate clinical method for measuring denture stomatitis severity.

Note for 4.2: The same clinical examination and Newton's classification criteria were used for participants in both intervention groups. Palatal photographs were also obtained to support diagnostic agreement among research team members.

Note for 4.3: The supplementary CONSORT chart states that the investigator was blinded. Therefore, the clinical outcome assessor was not aware of the intervention received by participants.

D5 Selection of the reported result

Signalling-question responses	Assessor judgement
5.1: PY; 5.2: N; 5.3: N	Low

Note for 5.1: The supplementary materials identify the clinical cure rate as the primary outcome. Participants were registered in a clinical trial record, and the study pre-specified clinical assessment using Newton's classification. It is probable that the reported clinical result followed a pre-specified plan.

Note for 5.2: Clinical outcomes were assessed at baseline and on days 7, 14 and 21. The result assessed here is from day 21, the final scheduled assessment after the full 3-week treatment period. There was no indication that this final clinical result was selected on the basis of its findings.

Note for 5.3: Clinical cure rate was identified as the primary outcome. The report used chi-square testing for clinical response between groups, with no indication that the final result was selected from multiple alternative analyses on the basis of the findings.

Overall judgement

Low

Sabzghabaee (2011) clinical outcome

Domains 1 to 3

Intervention: Pelargonium graveolens 1% Orabase gel, twice daily for 14 days

Comparator: Placebo Orabase gel, twice daily for 14 days

Outcome assessed: Clinical outcome: clinical recovery of denture stomatitis (palatal erythema) at 14 days

Result assessed: Clinical recovery of denture stomatitis (palatal erythema) at 14 days

Evidence sources recorded: Journal article(s)

D1 Randomization process

Signalling-question responses	Assessor judgement
1.1: Y; 1.2: PY; 1.3: PN	Low

Note for 1.1&1.2: Participants were reported to have been randomly divided into two groups using random-figure tables. The article states that allocation was based on participants' profile numbers and random-figure tables. Although the process is insufficiently described, this probably indicates that the random allocation sequence was applied after participant enrolment; no clear evidence indicates that allocation was predictable or manipulable.

Note for 1.3: The authors reported that the groups were comparable regarding sex, denture-use duration, denture-wearing habits, Newton's type of denture stomatitis and baseline palatal Candida colony count. Although mean age differed (58.85 versus 63.92 years; P=0.021), this isolated imbalance in a small trial does not probably indicate a problem with the randomization process.

D2 Deviations from intended interventions

Signalling-question responses	Assessor judgement
2.1: N; 2.2: N; 2.3: NA; 2.4: NA; 2.5: NA; 2.6: Y; 2.7: NA	Low

Note for 2.1&2.2: The study was described as double-blind. Participants received either 1% Pelargonium graveolens gel or a placebo consisting of the same gel base, which supports that participants were not aware of their assigned intervention. The study was described as double-blind. The intervention and placebo were prepared as comparable gel formulations, and there is no indication that the personnel delivering the intervention were aware of treatment allocation.

Note for 2.6: Clinical recovery outcomes were reported for all 80 randomized participants, with 40 participants in each group. No exclusions, losses to follow-up, crossovers or treatment discontinuations were reported; therefore, the analysis probably estimated the effect of assignment to intervention.

D3 Missing outcome data

Signalling-question responses	Assessor judgement
3.1: Y; 3.2: NA; 3.3: NA; 3.4: NA	Low

Note for 3.1: The study reports two groups of 40 participants and does not describe missing clinical outcome data, withdrawals or exclusions. The retained Y response relies on the reported group sizes and absence of reported attrition. However, the published clinical percentages do not reconcile with integer event counts out of 40; they cannot independently verify the analysed denominator.

Sabzghabaee (2011) clinical outcome

Domains 4 and 5

Outcome assessed: Clinical outcome: clinical recovery of denture stomatitis (palatal erythema) at 14 days

D4 Measurement of the outcome

Signalling-question responses	Assessor judgement
4.1: PN; 4.2: N; 4.3: N; 4.4: NA; 4.5: NA	Low

Note for 4.1: Recovery was an author-defined clinical–mycological outcome combining lesion assessment with fungal culture findings; it should not be interpreted as a purely clinical endpoint. Clinical examination was performed by a specialized dentist, and samples were cultured on Sabouraud dextrose agar, incubated at 37°C for 48 hours and assessed by colony counting. The laboratory component provides an objective element but does not eliminate subjectivity in visual lesion assessment. Validation and calibration of the clinical assessment were not reported (pp. S106–S107).

Note for 4.2: The same clinical examination and culture procedures were described for both groups, and the reported recovery categories were applied to the intervention and placebo groups. No differential ascertainment procedure between groups was described (pp. S106–S107).

Note for 4.3: The trial was described as double-blind and used the same gel base for the active and placebo formulations. The retained response interprets the reported double-blind design as covering outcome assessment; however, the identity of each blinded assessor and the success of blinding were not explicitly documented. The low-risk judgement considers both components of recovery and does not rely on colony counting alone (pp. S106–S107).

D5 Selection of the reported result

Signalling-question responses	Assessor judgement
5.1: NI; 5.2: N; 5.3: N	Some concerns

Note for 5.1: No trial protocol, statistical analysis plan or prospective trial registry record was available. Therefore, it was not possible to determine whether the analysis of clinical recovery at day 14 followed a pre-specified analysis plan finalized before unblinded outcome data were available.

Note for 5.2: Clinical status was assessed at baseline and on days 7 and 14. The reported result is the day-14 outcome, corresponding to the end of the pre-specified 14-day treatment course. There is no indication that this time point was selected on the basis of the results.

Note for 5.3: The article reported the predefined categories of complete, partial and no improvement, analysed qualitative outcomes using the chi-square test. There is no indication that the reported clinical recovery result was selected from multiple eligible analyses on the basis of the findings.

Overall judgement

Some concerns

Sabzghabaee (2012) clinical outcome

Domains 1 to 3

Intervention: Satureja hortensis essential oil 1% gel, twice daily for 14 days

Comparator: Placebo gel, twice daily for 14 days

Outcome assessed: Clinical outcome: Clinical recovery of denture stomatitis (palatal erythema) at 14 days

Result assessed: Complete or partial clinical improvement: 87.5% (35/40) versus 30.0% (12/40)

Evidence sources recorded: Journal article(s)

D1 Randomization process

Signalling-question responses	Assessor judgement
1.1: Y; 1.2: PY; 1.3: N	Low

Note for 1.1&1.2: The article states that patients were randomly assigned to the 1% Satureja hortensis gel or placebo group using their profile numbers and random-figure tables. Allocation was reported to be based on participants' profile numbers and random-figure tables. Although the concealment procedure was not fully described, there is no evidence that the allocation sequence was predictable or manipulable.

Note for 1.3: The groups were similar at baseline for age, sex, denture-use duration, Newton's type of denture stomatitis, overnight denture removal and baseline Candida colony count. No statistically significant baseline differences were reported.

D2 Deviations from intended interventions

Signalling-question responses	Assessor judgement
2.1: N; 2.2: N; 2.3: NA; 2.4: NA; 2.5: NA; 2.6: Y; 2.7: NA	Low

Note for 2.1&2.2: The trial was described as double-blind. Participants received either 1% Satureja hortensis essential-oil gel or placebo formulated from the same gel base; therefore, participants were not aware of their assigned intervention. The study was described as double-blind. The intervention and placebo used the same gel base, and there was no indication that personnel delivering the gels were aware of treatment allocation.

Note for 2.6: The report states that all 80 participants completely fulfilled the study protocol. Clinical recovery was reported for both randomized groups of 40 participants, with no withdrawals, exclusions, treatment discontinuations or crossovers reported.

D3 Missing outcome data

Signalling-question responses	Assessor judgement
3.1: Y; 3.2: NA; 3.3: NA; 3.4: NA	Low

Note for 3.1: The report states that all 80 participants completely fulfilled the study protocol. Clinical recovery data were reported for both randomized groups of 40 participants, and no missing outcome data, withdrawals or exclusions were reported.

Sabzghabaee (2012) clinical outcome

Domains 4 and 5

Outcome assessed: Clinical outcome: Clinical recovery of denture stomatitis (palatal erythema) at 14 days

D4 Measurement of the outcome

Signalling-question responses	Assessor judgement
4.1: NI; 4.2: N; 4.3: PN; 4.4: NA; 4.5: NA	Low

Note for 4.1: Recovery was an author-defined clinical–mycological outcome combining lesion assessment with fungal culture findings; it should not be interpreted as a purely clinical endpoint. Clinical examination was performed by a specialized dentist, and samples were cultured on Sabouraud dextrose agar, incubated at 37°C for 48 hours and assessed by colony counting. The laboratory component provides an objective element but does not eliminate subjectivity in visual lesion assessment. Validation and calibration of the clinical assessment were not reported (pp. 199–200).

Note for 4.2: The same clinical examination and culture procedures were described for both groups, and the reported recovery categories were applied to the intervention and placebo groups. No differential ascertainment procedure between groups was described (pp. 199–200).

Note for 4.3: The trial was described as double-blind and used the same gel base for the active and placebo formulations. The retained response interprets the reported double-blind design as covering outcome assessment; however, the identity of each blinded assessor and the success of blinding were not explicitly documented. The low-risk judgement considers both components of recovery and does not rely on colony counting alone (pp. 199–200).

D5 Selection of the reported result

Signalling-question responses	Assessor judgement
5.1: NI; 5.2: N; 5.3: N	Some concerns

Note for 5.1: No trial protocol, statistical analysis plan or prospective trial registry record was available. Therefore, it was not possible to determine whether the analysis of clinical recovery at day 14 followed a pre-specified analysis plan finalized before unblinded outcome data were available.

Note for 5.2: Clinical and mycological evaluations were conducted at baseline and repeated after 14 days, the end of the planned treatment course. There is no indication that the reported day-14 clinical recovery result was selected on the basis of its findings.

Note for 5.3: The article describes the use of chi-square tests for qualitative outcomes and reports the predefined categories of complete, partial and no improvement. There is no indication that the reported clinical recovery result was selected from multiple eligible analyses on the basis of the findings

Overall judgement

Some concerns

Sefidgar (2010) clinical outcome

Domains 1 to 3

Intervention: Artemisia sieberi 1% mouthwash, 20 mL four times daily

Comparator: Nystatin mouthwash, 500,000 IU four times daily

Outcome assessed: Time to complete clinical resolution of denture stomatitis

Result assessed: Complete clinical resolution: 14/15 in each group after 1 week; 15/15 in each group after 2 weeks

Evidence sources recorded: Journal article(s)

D1 Randomization process

Signalling-question responses	Assessor judgement
1.1: PY; 1.2: NI; 1.3: PN	Some concerns

Note for 1.1&1.2: The article states that patients were randomly divided into intervention and control groups, but it does not describe the method used to generate the allocation sequence. The article does not report how allocation was concealed until participants were enrolled and assigned to the Artemisia or nystatin group. Howewver it states that the study is blinded.

Note for 1.3: The groups had identical distributions of Newton type II and III denture stomatitis (9 and 6 participants, respectively). Age and sex distributions were also similar, with no apparent baseline imbalance suggesting a problem with randomization.

D2 Deviations from intended interventions

Signalling-question responses	Assessor judgement
2.1: N; 2.2: NI; 2.3: N; 2.4: NA; 2.5: NA; 2.6: Y; 2.7: NA	Low

Note for 2.1&2.2: The article states that patients were blinded to what they received and to whether they were in the intervention or control group. The article reported blinding of patients and the clinical examiner, but it did not state whether carers or personnel who dispensed or delivered the mouthwashes were aware of treatment allocation.

Note for 2.6: All 30 patients completed the treatment course. Clinical healing was reported for all 15 participants in each group, with no withdrawals, exclusions, treatment discontinuations or crossovers reported.

D3 Missing outcome data

Signalling-question responses	Assessor judgement
3.1: Y; 3.2: NA; 3.3: NA; 3.4: NA	Low

Note for 3.1: All 30 patients completed the treatment course. Clinical healing data were reported for all 15 participants in each group: 14 per group achieved complete resolution after one week and the remaining participant in each group achieved complete resolution after two weeks.

Sefidgar (2010) clinical outcome

Domains 4 and 5

Outcome assessed: Time to complete clinical resolution of denture stomatitis

D4 Measurement of the outcome

Signalling-question responses	Assessor judgement
4.1: NI; 4.2: PN; 4.3: N; 4.4: NA; 4.5: NA	Low

Note for 4.1: Clinical healing was assessed at weekly visits, but the article did not describe a validated clinical scoring system, explicit criteria for complete clinical resolution, examiner calibration, standardized photographs or independent outcome adjudication. Therefore, there is no information to determine whether the method of measuring clinical healing was appropriate.

Note for 4.2: Clinical healing was assessed at the same weekly visits in both groups, and no group-specific outcome measurement procedure was described

Note for 4.3: The article explicitly states that the clinical examiner was blinded to what patients received and to whether they were in the intervention or control group.

D5 Selection of the reported result

Signalling-question responses	Assessor judgement
5.1: PN; 5.2: PN; 5.3: PN	Some concerns

Note for 5.1: No protocol, statistical analysis plan or prospective registration record was available to verify a plan finalized before unblinded outcome data were available. The retained PN response reflects the assessor inference that a prespecified analysis plan was probably not available; it is not direct evidence that analyses were selected after inspecting results.

Note for 5.2: Several weekly assessments were reported, with follow-up until one week after clinical resolution. No fixed primary analysis time point could be verified against a protocol. The retained PN response reflects the assessor judgement that selection among measurements on the basis of the results was probably absent, although this could not be independently verified.

Note for 5.3: Friedman, chi-square and Mann–Whitney tests were mentioned, but no analysis plan was available. The retained PN response reflects the assessor judgement that result-dependent selection among eligible analyses was probably absent. The existence of several tests does not establish selective reporting, but prespecification cannot be confirmed.

Overall judgement

Some concerns

Amanlou (2006) mycological outcome

Domains 1 to 3

Intervention: Zataria multiflora 0.1% essential oil gel, 4 times daily for 14 days

Comparator: Miconazole 2% gel, 4 times daily for 14 days

Outcome assessed: Microbiological outcome: Candida colony count from palatal mucosa at day 14

Result assessed: Mean colony-count score at day 14; Figure 2

Evidence sources recorded: Journal article(s)

D1 Randomization process

Signalling-question responses	Assessor judgement
1.1: PY; 1.2: PY; 1.3: N	Low

Note for 1.1&1.2: Participants were reported to be randomly assigned to the miconazole or Z. multiflora gel group; however, the method used to generate the random allocation sequence was not described.

Note for 1.3: The groups were reported to be similar at baseline with respect to age, sex, systemic disease history, denture-wearing characteristics, Candida colony counts and erythema surface

D2 Deviations from intended interventions

Signalling-question responses	Assessor judgement
2.1: N; 2.2: N; 2.3: NA; 2.4: NA; 2.5: NA; 2.6: Y; 2.7: NA	Low

Note for 2.1&2.2: The study was reported as double-blind, and the appearance, colour, smell and taste of the two gels were made as similar as possible. The study was reported as double-blind, and treatment codes were not known during the clinical examination

Note for 2.6: All 24 randomized participants were reported in two groups of 12, and no exclusions from analysis were reported.

D3 Missing outcome data

Signalling-question responses	Assessor judgement
3.1: Y; 3.2: NA; 3.3: NA; 3.4: NA	Low

Note for 3.1: No missing microbiological outcome data or exclusions from the analysis were reported.

Amanlou (2006) mycological outcome

Domains 4 and 5

Outcome assessed: Microbiological outcome: Candida colony count from palatal mucosa at day 14

D4 Measurement of the outcome

Signalling-question responses	Assessor judgement
4.1: N; 4.2: N; 4.3: N; 4.4: NA; 4.5: NA	Low

Note for 4.1: Candida samples were collected using sterile cotton swabs, cultured on Sabouraud dextrose agar, incubated for 48 hours, and evaluated using a predefined culture-density rating scale; the method was appropriate for this outcome

Note for 4.2: The same sampling, culture and outcome-assessment procedures were applied to both intervention groups.

Note for 4.3: The study was reported as double-blind, and treatment codes were not known during the examination.

D5 Selection of the reported result

Signalling-question responses	Assessor judgement
5.1: NI; 5.2: N; 5.3: N	Some concerns

Note for 5.1: No pre-specified analysis plan, protocol, statistical analysis plan, or trial registration record was available.

Note for 5.2: Candida culture-density was assessed at several post-baseline time points; however, results for all reported follow-up visits were presented. There was no indication that the day 14 result was selected on the basis of its findings

Note for 5.3: The report suggests a single standard between-group analysis for the outcome. No multiple adjusted, subgroup, or alternative analyses were reported; however, the analysis details were limited.

Overall judgement

Some concerns

Araujo (2021) mycological outcome

Domains 1 to 3

Intervention: Cinnamomum zeylanicum essential oil 0.5 mg/mL mouthwash and denture spray, 3 times daily for 15 days

Comparator: Nystatin 100,000 IU/mL, 3 times daily for 15 days

Outcome assessed: Microbiological outcome: reduction in Candida CFU from oral mucosa at day 16

Result assessed: Participants with a reduction in Candida CFU: 11/18 versus 16/18; Results section

Evidence sources recorded: Journal article(s); Non-commercial trial registry record (e.g. ClinicalTrials.gov record)

D1 Randomization process

Signalling-question responses	Assessor judgement
1.1: Y; 1.2: Y; 1.3: N	Low

Note for 1.1&1.2: The allocation sequence was generated using a computer program. Allocation concealment was ensured by an individual who was not one of the study investigators.

Note for 1.3: The groups were reported to be homogeneous at baseline with respect to clinical, mycological and denture-use characteristics.

D2 Deviations from intended interventions

Signalling-question responses	Assessor judgement
2.1: N; 2.2: N; 2.3: NA; 2.4: NA; 2.5: NA; 2.6: Y; 2.7: NA	Low

Note for 2.1&2.2: The trial was double-blind. The study products had the same colour, taste and odour and were stored in identical dark vials labelled A or B. Investigators involved in the clinical stage were unaware of treatment allocation. The investigator responsible for randomization and the pharmacist preparing the products did not participate in the clinical stage.

Note for 2.6: All 36 randomized participants were included in the reported analysis, with 18 participants in each intervention group.

D3 Missing outcome data

Signalling-question responses	Assessor judgement
3.1: Y; 3.2: NA; 3.3: NA; 3.4: NA	Low

Note for 3.1: Data on reduction in Candida CFU from the oral mucosa were reported for all randomized participants: 11/18 in the C. zeylanicum group and 16/18 in the nystatin group.

Araujo (2021) mycological outcome

Domains 4 and 5

Outcome assessed: Microbiological outcome: reduction in Candida CFU from oral mucosa at day 16

D4 Measurement of the outcome

Signalling-question responses	Assessor judgement
4.1: N; 4.2: N; 4.3: N; 4.4: NA; 4.5: NA	Low

Note for 4.1: Candida CFU were measured from oral mucosal swabs cultured on CHROMagar Candida for 48 hours. The culture and CFU-counting method was appropriate for this microbiological outcome.

Note for 4.2: Oral mucosal samples were collected and processed using the same swab, culture and CFU-counting procedures in both intervention groups.

Note for 4.3: The clinical investigators were reported to be blinded to treatment allocation throughout the clinical stage

D5 Selection of the reported result

Signalling-question responses	Assessor judgement
5.1: PY; 5.2: N; 5.3: N	Low

Note for 5.1: The trial registry specified microbiological assessment of Candida CFU from the affected oral mucosa as a secondary outcome. The published report presented microbiological results for this outcome at the planned end of the 15-day intervention.

Note for 5.2: Mycological samples were collected at baseline and at the planned post-treatment visit, 24 hours after the final dose of the 15-day intervention. No indication of result-dependent selection among post-treatment time points was identified.

Note for 5.3: The assessed result was the proportion of participants with a reduction in mucosal Candida counts, reported for both randomized groups. No multiple adjusted, subgroup or alternative analyses of that response were described. The retained N response reflects the absence of an indication that this result was selected from multiple eligible analyses on the basis of its findings.

Overall judgement

Low

Catalan (2008) mycological outcome

Domains 1 to 3

Intervention: Melaleuca alternifolia oil mixed with Coe-Comfort tissue conditioner, applied as a denture reline and replaced every 4 days for 12 days

Comparator: Nystatin mixed with Coe-Comfort tissue conditioner, applied as a denture reline and replaced every 4 days for 12 days

Outcome assessed: Microbiological outcome: Mean log10 Candida albicans CFU from palatal mucosa at day 12

Result assessed: No statistically significant difference in mean log10 Candida albicans CFU between the Melaleuca alternifolia and nystatin groups at the final session (day 12; P = 0.77).

Evidence sources recorded: Journal article(s)

D1 Randomization process

Signalling-question responses	Assessor judgement
1.1: PY; 1.2: NI; 1.3: PN	Some concerns

Note for 1.1&1.2: The report states that patients were randomly divided into three 9-person treatment groups, but it does not describe how the random allocation sequence was generated. The report provides no information on allocation concealment.

Note for 1.3: Baseline characteristics were described for the full study sample, but baseline data were not reported separately for the three randomized groups.

D2 Deviations from intended interventions

Signalling-question responses	Assessor judgement
2.1: NI; 2.2: NI; 2.3: PN; 2.4: NA; 2.5: NA; 2.6: Y; 2.7: NA	Low

Note for 2.1&2.2: The report does not state whether participants were blinded to the intervention received. The report does not state whether the clinicians preparing and applying the tissue conditioners were blinded to treatment allocation.

Note for 2.3: he interventions were applied and replaced by the study team at scheduled clinical visits. No deviations from the assigned treatment, co-interventions or protocol departures were reported; therefore, deviations arising because of the trial context were probably absent.

Note for 2.6: Microbiological outcomes were analysed using repeated-measures ANOVA, and culture results were reported for the scheduled visits in the randomized treatment groups.

D3 Missing outcome data

Signalling-question responses	Assessor judgement
3.1: Y; 3.2: NA; 3.3: NA; 3.4: NA	Low

Note for 3.1: Mycological culture results were reported for the three randomized groups at all four scheduled sessions: baseline and days 4, 8 and 12. No missing microbiological data, exclusions or losses to follow-up were reported.

Catalan (2008) mycological outcome

Domains 4 and 5

Outcome assessed: Microbiological outcome: Mean log10 Candida albicans CFU from palatal mucosa at day 12

D4 Measurement of the outcome

Signalling-question responses	Assessor judgement
4.1: N; 4.2: N; 4.3: NI; 4.4: PN; 4.5: NA	Low

Note for 4.1: Candida albicans was measured from palatal mucosal swabs using culture on Sabouraud agar, incubation for 48 hours and CFU counting. This was an appropriate method for the microbiological outcome.

Note for 4.2: Samples were collected from the palatal mucosa and processed using the same swab, transport, culture, incubation and CFU-counting procedures in all treatment groups.

Note for 4.3: The report does not state whether laboratory personnel counting Candida albicans CFU were blinded to treatment allocation.

Note for 4.4&4.5: CFU counting after standardized culture is an objective laboratory measurement. Knowledge of treatment allocation was probably not able to influence the measured colony count.

D5 Selection of the reported result

Signalling-question responses	Assessor judgement
5.1: NI; 5.2: N; 5.3: N	Some concerns

Note for 5.1: No trial protocol, prospective trial registration record or statistical analysis plan was available. Therefore, it was not possible to determine whether the analysis followed a pre-specified plan finalized before unblinded outcome data were available.

Note for 5.2: Candida albicans CFU were measured at baseline and on days 4, 8 and 12. The reported result for this assessment relates to day 12, the final scheduled examination at the end of the 12-day treatment period. There was no indication that this end-of-treatment result was selected on the basis of its findings.

Note for 5.3: The report described one repeated-measures ANOVA approach for the microbiological outcome, with multiple comparisons when appropriate. There was no indication that the final-session result was selected from multiple alternative analyses on the basis of the findings.

Overall judgement

Some concerns

Gonoudi (2021) mycological outcome

Domains 1 to 3

Intervention: Zataria multiflora essential oil 0.05%, 5 mL mouth rinse for 2 minutes, four times daily for 14 days

Comparator: Nystatin suspension 100,000 units, 40 drops mouth rinse for 2 minutes, four times daily for 14 days

Outcome assessed: Mycological outcome: Candida albicans colony count (CFU) from palatal swab at day 14

Result assessed: Mean Candida albicans CFU at day 14: 19,071.43 ± 28,089.04 in the Zataria multiflora group versus 22,000.00 ± 32,825.88 in the nystatin group; between-group P = 0.593.

Evidence sources recorded: Journal article(s)

D1 Randomization process

Signalling-question responses	Assessor judgement
1.1: Y; 1.2: Y; 1.3: N	Low

Note for 1.1&1.2: Stratified block randomization was used according to denture stomatitis type II or III. Allocation was based on computer-generated random numbers. Medications were dispensed by a person who was not involved in the study, according to the computer-generated random allocation sequence.

Note for 1.3: The groups were balanced for denture stomatitis type, with seven participants with type II and seven with type III stomatitis in each group. Baseline demographic and clinical characteristics showed no statistically significant between-group differences.

D2 Deviations from intended interventions

Signalling-question responses	Assessor judgement
2.1: NI; 2.2: PN; 2.3: PN; 2.4: NA; 2.5: NA; 2.6: Y; 2.7: NA	Low

Note for 2.1&2.2: The study was described as single-blind, but the report did not specify whether participants were blinded to the intervention received. Study medications were dispensed by another person according to the computer-generated random allocation sequence. This person had to know the assigned intervention in order to dispense either Zataria multiflora or nystatin.

Note for 2.3: co-interventions were reported; deviations arising because of the trial context were probably absent.

Note for 2.6: All 28 randomized participants were included in the two study groups, with 14 participants per group. The study reported between-group analysis of Candida albicans colony counts using repeated-measures ANOVA.

D3 Missing outcome data

Signalling-question responses	Assessor judgement
3.1: Y; 3.2: NA; 3.3: NA; 3.4: NA	Low

Note for 3.1: Candida albicans colony count data before and after treatment were reported for all 28 randomized participants, with 14 participants in the Zataria multiflora group and 14 participants in the nystatin group. No losses to follow-up or exclusions from the microbiological outcome analysis were reported.

Gonoudi (2021) mycological outcome

Domains 4 and 5

Outcome assessed: Mycological outcome: Candida albicans colony count (CFU) from palatal swab at day 14

D4 Measurement of the outcome

Signalling-question responses	Assessor judgement
4.1: N; 4.2: N; 4.3: NI; 4.4: PN; 4.5: NA	Low

Note for 4.1: Candida albicans CFU were measured from palatal swabs using standardized culture on Sabouraud agar, incubation, germ tube confirmation and colony counting under a light microscope. This was an appropriate method for the mycological outcome.

Note for 4.2: The same palatal swab collection, transport, culture, incubation, Candida albicans confirmation and CFU-counting procedures were used in both intervention groups.

Note for 4.3: The study was described as single-blind, but the report did not specify whether laboratory personnel assessing Candida albicans CFU were blinded to treatment allocation.

Note for 4.4&4.5: Candida albicans colony counting after standardized culture and germ tube confirmation is an objective laboratory measurement. Knowledge of treatment allocation was probably not able to materially influence the measured CFU count.

D5 Selection of the reported result

Signalling-question responses	Assessor judgement
5.1: PY; 5.2: N; 5.3: N	Low

Note for 5.1: The report states that the trial was registered in the Iranian Registry of Clinical Trials in 2017. Candida albicans colony count was specified as an outcome and was assessed at baseline and after the planned 14-day treatment period. It is probable that the reported analysis followed a pre-specified plan.

Note for 5.2: Candida albicans CFU were measured at baseline and at one planned post-treatment assessment on day 14. There was no indication that the reported day 14 result was selected from multiple eligible post-treatment time points on the basis of its findings.

Note for 5.3: The report described repeated-measures ANOVA for the Candida albicans colony-count outcome. There was no indication that the reported day 14 result was selected from multiple alternative analyses on the basis of the findings.

Overall judgement

Low

Iyer (2022) mycological outcome

Domains 1 to 3

Intervention: Optimized clove/cinnamon supercritical fluid extracts-loaded emulgel (clove:cinnamon extract ratio 1:8)

Comparator: Commercially available antifungal gel (Candid® gel)

Outcome assessed: Mycological outcome: fungal colony count (CFU/mL) from palatal surface at day 21

Result assessed: Mean fungal colony count at day 21 from the palatal surface: 91.76 ± 61.07 CFU/mL in the clove/cinnamon emulgel group versus 66.38 ± 39.11 CFU/mL in the Candid® gel group.

Evidence sources recorded: Journal article(s)

D1 Randomization process

Signalling-question responses	Assessor judgement
1.1: Y; 1.2: Y; 1.3: N	Low

Note for 1.1&1.2: The supplementary materials state that randomization was performed using computer-generated random numbers. The supplementary CONSORT chart states that both the investigator and study participants were blinded. Medication tubes were coded and distributed according to participant number and group allocation, supporting concealment of allocation during enrolment and assignment.

Note for 1.3: The groups had similar mean age and gender distribution. Baseline Newton's classification was broadly comparable and did not suggest a problem with randomization.

D2 Deviations from intended interventions

Signalling-question responses	Assessor judgement
2.1: N; 2.2: N; 2.3: NA; 2.4: NA; 2.5: NA; 2.6: Y; 2.7: NA	Low

Note for 2.1&2.2: The supplementary CONSORT chart states that study participants were blinded. Medication tubes were coded, so participants were not aware of the assigned intervention. The supplementary CONSORT chart states that the investigator was blinded. Study medications were supplied in coded tubes, and participants applied the assigned gel themselves according to the same written and verbal protocol

Note for 2.6: Fungal colony counts were analysed using repeated-measures ANOVA, and CFU data at day 21 were reported for all 42 randomized participants, with 21 participants in each group.

D3 Missing outcome data

Signalling-question responses	Assessor judgement
3.1: Y; 3.2: NA; 3.3: NA; 3.4: NA	Low

Note for 3.1: Fungal colony count data from the palatal surface at day 21 were reported for both randomized groups. Each group contained 21 participants, and no losses to follow-up, missing mycological data or exclusions from analysis were reported.

Iyer (2022) mycological outcome

Domains 4 and 5

Outcome assessed: Mycological outcome: fungal colony count (CFU/mL) from palatal surface at day 21

D4 Measurement of the outcome

Signalling-question responses	Assessor judgement
4.1: N; 4.2: N; 4.3: N; 4.4: NA; 4.5: NA	Low

Note for 4.1: Fungal burden was measured from palatal swabs using culture on Sabouraud dextrose agar, incubation for 48 hours and digital colony counting with ImageJ/OpenCFU software. This was an appropriate method for measuring the mycological outcome.

Note for 4.2: Palatal swabs from both intervention groups were collected, cultured, incubated and analysed using the same standardized laboratory and digital colony-counting procedures.

Note for 4.3: Supplementary material states that the investigators were blinded.

D5 Selection of the reported result

Signalling-question responses	Assessor judgement
5.1: PY; 5.2: N; 5.3: N	Low

Note for 5.1: The supplementary materials identify fungal burden reduction rate as a secondary outcome. Fungal CFU from palatal and denture surfaces were measured at the planned follow-up visits. It is probable that the reported palatal fungal burden result followed a pre-specified plan.

Note for 5.2: Fungal burden was measured at baseline and on days 7, 14 and 21. The result assessed here is from day 21, the final scheduled assessment after the complete 3-week treatment period. There was no indication that this final result was selected on the basis of its findings.

Note for 5.3: The report described repeated-measures ANOVA for CFU/mL reduction and presented CFU results across the planned time points. There was no indication that the day 21 palatal CFU result was selected from multiple alternative analyses on the basis of the findings.

Overall judgement

Low

Sabzghabaee (2011) mycological outcome

Domains 1 to 3

Intervention: Pelargonium graveolens 1% Orabase gel, twice daily for 14 days

Comparator: Placebo Orabase gel, twice daily for 14 days

Outcome assessed: Mycological outcome: Candida albicans colony count from palatal mucosa at 14 days

Result assessed: Mean colony count: 9.30 (SD 10.87) versus 26.20 (SD 27.89) CFU per sample

Evidence sources recorded: Journal article(s)

D1 Randomization process

Signalling-question responses	Assessor judgement
1.1: Y; 1.2: PY; 1.3: PN	Low

Note for 1.1&1.2: Participants were reported to have been randomly divided into two groups using random-figure tables. The article states that allocation was based on participants' profile numbers and random-figure tables. Although the process is insufficiently described, this probably indicates that the random allocation sequence was applied after participant enrolment; no clear evidence indicates that allocation was predictable or manipulable.

Note for 1.3: The authors reported that the groups were comparable regarding sex, denture-use duration, denture-wearing habits and Newton's type of denture stomatitis. Although baseline colony counts differed numerically (38.05 versus 30.40 CFU per sample), the reported random allocation method and the absence of a consistent pattern of baseline imbalances probably do not suggest a problem with the randomization process.

D2 Deviations from intended interventions

Signalling-question responses	Assessor judgement
2.1: N; 2.2: N; 2.3: NA; 2.4: NA; 2.5: NA; 2.6: Y; 2.7: NA	Low

Note for 2.1&2.2: The trial was described as double-blind. Participants received either 1% Pelargonium graveolens gel or placebo consisting of the same gel base; therefore, participants were not aware of their assigned intervention. The trial was described as double-blind, and the placebo was the same gel base without Pelargonium graveolens essential oil. There was no indication that the personnel involved in delivering the intervention were aware of treatment allocation.

Note for 2.6: Colony-count results after treatment were reported for both randomized groups of 40 participants. No exclusions, withdrawals, treatment discontinuations, crossovers or missing outcome data were reported; therefore, the analysis probably estimated the effect of assignment to intervention.

D3 Missing outcome data

Signalling-question responses	Assessor judgement
3.1: Y; 3.2: NA; 3.3: NA; 3.4: NA	Low

Note for 3.1: Mycological outcome data after treatment were reported for both randomized groups, each comprising 40 participants. The article did not report missing fungal culture data, withdrawals, exclusions or losses to follow-up.

Sabzghabaee (2011) mycological outcome

Domains 4 and 5

Outcome assessed: Mycological outcome: Candida albicans colony count from palatal mucosa at 14 days

D4 Measurement of the outcome

Signalling-question responses	Assessor judgement
4.1: N; 4.2: N; 4.3: N; 4.4: NA; 4.5: NA	Low

Note for 4.1: Candida albicans colony counts were measured from palatal swabs using culture on Sabouraud dextrose agar, incubation at 37°C for 48 hours and colony counting. This was an appropriate method for measuring the mycological outcome.

Note for 4.2: Palatal samples from both intervention groups were collected at the same scheduled visits and were processed using the same culture medium, incubation conditions and colony-counting procedure.

Note for 4.3: Study described as double blind.

D5 Selection of the reported result

Signalling-question responses	Assessor judgement
5.1: NI; 5.2: N; 5.3: N	Some concerns

Note for 5.1: No trial protocol, statistical analysis plan or prospective trial registry record was available. Therefore, it was not possible to determine whether the analysis of Candida colony counts at day 14 followed a pre-specified analysis plan finalized before unblinded outcome data were available.

Note for 5.2: Mycological samples were collected at baseline and on days 7 and 14. The reported colony-count result is from day 14, corresponding to the end of the planned 14-day treatment course. There is no indication that this time point was selected on the basis of the results.

Note for 5.3: The article states that quantitative data were analysed using Student’s t-test and reports mean colony counts with standard deviations for each group. There is no indication that the reported day-14 colony-count result was selected from multiple eligible analyses on the basis of the findings.

Overall judgement

Some concerns

Sabzghabaee (2012) mycological outcome

Domains 1 to 3

Intervention: Satureja hortensis essential oil 1% gel, twice daily for 14 days

Comparator: Placebo gel, twice daily for 14 days

Outcome assessed: Mycological outcome: Candida albicans colony count from palatal mucosa at 14 days

Result assessed: Mean colony count: 10.00 (SD 16.148) versus 26.20 (SD 27.886) CFU per sample

Evidence sources recorded: Journal article(s)

D1 Randomization process

Signalling-question responses	Assessor judgement
1.1: Y; 1.2: PY; 1.3: N	Low

Note for 1.1&1.2: Patients were reported to have been randomly assigned to 1% Satureja hortensis gel or placebo using their profile numbers and random-figure tables. Allocation was based on participants' profile numbers and random-figure tables. Although the concealment procedure was not fully described, there is no evidence that allocation was predictable or manipulable.

Note for 1.3: The groups were similar at baseline for age, sex, denture-use duration, Newton's type of denture stomatitis, overnight denture removal and baseline Candida colony count. No statistically significant baseline differences were reported.

D2 Deviations from intended interventions

Signalling-question responses	Assessor judgement
2.1: N; 2.2: N; 2.3: NA; 2.4: NA; 2.5: NA; 2.6: Y; 2.7: NA	Low

Note for 2.1&2.2: The trial was described as double-blind. Participants received either 1% Satureja hortensis essential-oil gel or placebo formulated from the same gel base; therefore, participants were not aware of their assigned intervention. The study was described as double-blind. The intervention and placebo used the same gel base, and there was no indication that personnel delivering the gels were aware of treatment allocation.

Note for 2.6: All 80 participants completely fulfilled the study protocol. Mycological outcome data after treatment were reported for both randomized groups of 40 participants, with no withdrawals, exclusions, treatment discontinuations or crossovers reported.

D3 Missing outcome data

Signalling-question responses	Assessor judgement
3.1: Y; 3.2: NA; 3.3: NA; 3.4: NA	Low

Note for 3.1: The report states that all 80 participants completely fulfilled the study protocol. Mycological outcome data after treatment were reported for both randomized groups of 40 participants, with no missing fungal culture data, withdrawals or exclusions reported.

Sabzghabaee (2012) mycological outcome

Domains 4 and 5

Outcome assessed: Mycological outcome: Candida albicans colony count from palatal mucosa at 14 days

D4 Measurement of the outcome

Signalling-question responses	Assessor judgement
4.1: N; 4.2: N; 4.3: PN; 4.4: NA; 4.5: NA	Low

Note for 4.1: Candida albicans colony counts were measured from palatal mucosal swabs by culture on Sabouraud dextrose agar, incubation at 37°C for 48 hours and colony counting. This was an appropriate method for measuring the mycological outcome.

Note for 4.2: Palatal swabs from both groups were collected at the same study visits and processed using the same culture medium, incubation conditions and colony-counting procedure.

Note for 4.3: Trial described as double blind.

D5 Selection of the reported result

Signalling-question responses	Assessor judgement
5.1: NI; 5.2: N; 5.3: N	Some concerns

Note for 5.1: No trial protocol, statistical analysis plan or prospective trial registry record was available. Therefore, it was not possible to determine whether the analysis of Candida colony counts at day 14 followed a pre-specified analysis plan finalized before unblinded outcome data were available.

Note for 5.2: Mycological assessment was conducted at baseline and repeated after 14 days, corresponding to the end of the planned treatment course. There is no indication that the reported day-14 colony-count result was selected on the basis of its findings.

Note for 5.3: The report states that quantitative data were analysed using Student's t-test and reports mean colony counts with standard deviations for both groups. There is no indication that the reported day-14 colony-count result was selected from multiple eligible analyses on the basis of the findings.

Overall judgement

Some concerns

Sefidgar (2010) mycological outcome

Domains 1 to 3

Intervention: Artemisia sieberi 1% mouthwash, 20 mL four times daily

Comparator: Nystatin mouthwash, 500,000 IU four times daily

Outcome assessed: Microbiological outcome: Candida colony-growth score from palatal samples during treatment

Result assessed: Candida colony growth score at week 1: no growth in 3/15 (Artemisia) versus 14/15 (nystatin); at week 2: no growth in 15/15 in both groups

Evidence sources recorded: Journal article(s)

D1 Randomization process

Signalling-question responses	Assessor judgement
1.1: PY; 1.2: NI; 1.3: PN	Some concerns

Note for 1.1&1.2: The article states only that patients were randomly divided into two groups. No method for generating the allocation sequence was reported. The article did not report how allocation was concealed until participants were enrolled and assigned to the Artemisia or nystatin group.

Note for 1.3: The groups had identical distributions of Newton type II and III denture stomatitis and broadly similar age and sex distributions. There was no apparent baseline imbalance suggesting a problem with randomization.

D2 Deviations from intended interventions

Signalling-question responses	Assessor judgement
2.1: N; 2.2: NI; 2.3: N; 2.4: NA; 2.5: NA; 2.6: Y; 2.7: NA	Low

Note for 2.1&2.2: The article states that patients were blinded to what they received and to whether they were in the intervention or control group. The article reported blinding of patients and the clinical examiner, but it did not state whether personnel who dispensed or delivered the mouthwashes were aware of treatment allocation.

Note for 2.6: All 30 patients completed the treatment course. Candida colony-growth scores were reported for all 15 participants in each group at the study visits, with no withdrawals, exclusions, treatment discontinuations or crossovers reported.

D3 Missing outcome data

Signalling-question responses	Assessor judgement
3.1: Y; 3.2: NA; 3.3: NA; 3.4: NA	Low

Note for 3.1: The report states that all 30 patients completed treatment. Final colony-growth rows account for 15 participants in each group. Some earlier rows in Table 2 do not sum to the stated group size; this reporting inconsistency prevents confirmation of complete counts at every visit. The retained Y response relies on the reported completion and complete final-visit totals, not on consistent totals at all visits.

Sefidgar (2010) mycological outcome

Domains 4 and 5

Outcome assessed: Microbiological outcome: Candida colony-growth score from palatal samples during treatment

D4 Measurement of the outcome

Signalling-question responses	Assessor judgement
4.1: N; 4.2: N; 4.3: N; 4.4: NA; 4.5: NA	Low

Note for 4.1: Candida growth was assessed from palatal samples cultured on Sabouraud dextrose agar after incubation at 37°C for 72 hours. Colony growth was categorized using a predefined 0–4 scoring system, supported by smear microscopy and Candida identification tests. This was an appropriate method for measuring the microbiological outcome.

Note for 4.2: Palatal samples from both groups were collected at the same study visits and processed using the same culture medium, incubation conditions, colony-growth scoring system and identification procedures.

Note for 4.3: Study is stated to be double blind.

D5 Selection of the reported result

Signalling-question responses	Assessor judgement
5.1: PN; 5.2: PN; 5.3: PN	Some concerns

Note for 5.1: No protocol, statistical analysis plan or prospective registration record was available to verify a plan finalized before unblinded outcome data were available. The retained PN response reflects the assessor inference that a prespecified analysis plan was probably not available; it is not direct evidence that analyses were selected after inspecting results.

Note for 5.2: Several weekly assessments were reported, with follow-up until one week after clinical resolution. No fixed primary analysis time point could be verified against a protocol. The retained PN response reflects the assessor judgement that selection among measurements on the basis of the results was probably absent, although this could not be independently verified.

Note for 5.3: Friedman, chi-square and Mann–Whitney tests were mentioned, but no analysis plan was available. The retained PN response reflects the assessor judgement that result-dependent selection among eligible analyses was probably absent. The existence of several tests does not establish selective reporting, but prespecification cannot be confirmed.

Overall judgement

Some concerns
