# Supplementary material for: Essential Oils and Plant-Derived Volatile Compounds in the Treatment of Oral Candidiasis: A Systematic Review of Randomized Controlled Trials in Denture Stomatitis
Source: J Clin Med. 2026 Nov 5;15(21):8617. doi: 10.3390/jcm15218617 (PMC13764656; doi:10.3390/jcm15218617)
Supplement: Supplementary file 1 [file jcm-15-08617-s001.zip › Supplementary_Table_S4_safety.pdf]

Table S4. Safety, tolerability, and patient-reported outcomes in the included trials.

Study	Safety assessment method	Reported adverse effects	Tolerability and patient-reported outcomes
Amanlou et al. [24]	At each visit, mucosal examination for local reactions and questioning about adverse reactions.	Z. multiflora: itching, burning, vertigo, nausea, vomiting and bad taste; 59.3% reported in the original article (denominator unclear; see note *a). Miconazole: burning, vomiting and bad taste in 50% of participants.	Bad taste reported in both groups; separate taste assessment not described.
Araújo et al. [25]	Adverse effects recorded on clinical forms, including intensity, nature, onset, duration and timing over 15 days; clinical signs also investigated.	C. zeylanicum: 3/18 (16.7%): slight numbness (n = 2) and slight burning (n = 1). Nystatin: 1/18 (5.6%): tongue sensitivity.	Pleasant taste: C. zeylanicum, 15/18 (83.3%); nystatin, 11/18 (61.1%).
Catalán et al. [26]	Not reported.	Not reported.	Not reported.
Gonoudi et al. [27]	Not reported.	Not reported.	Not reported.
Iyer et al. [28]	Clinical adverse-event assessment method not described. Taste acceptability assessed using a hedonic scale.	Authors stated that the emulgel had no side effects; detailed clinical adverse-event data not provided.	Greater taste acceptability with the emulgel: 81% liked the test formulation; 76.2% disliked the clotrimazole gel.
Sabzghabaee et al., P. graveolens [29]	Adverse-event assessment method not described.	Authors reported no reportable side effects in participants receiving P. graveolens gel; comparator safety data not reported separately.	Not reported.
Sabzghabaee et al., S. hortensis [30]	On days 14 and 21, patients were assessed for local adverse reactions and questioned about adverse reactions.	No other reportable side effects observed in the herbal-gel group apart from the odor complaint; comparator safety data not reported separately.	Spicy odor was the only minor complaint; frequency not reported.
Sefidgar et al. [31]	Formal adverse-event or taste-assessment method not described.	No complaints reported in the Artemisia group; a structured adverse-event breakdown was not provided.	Artemisia taste described as desirable; one participant in the nystatin group reported undesirable taste.

Notes:

Reference numbers follow the supplied manuscript.

Not reported indicates that the publication did not provide the relevant information; it does not indicate that no adverse effects occurred. Statements that no side effects were observed are reproduced as author-reported findings and do not establish safety when assessment methods were not described.

*a The original Amanlou report states 59.3% in a group of 12 participants but provides no numerator or explanation of the denominator. This percentage cannot be reconciled with the reported group size and should not be converted to a participant count.
